# *In Silico* identification of SNP diversity in cultivated and wild tomato species: insight from molecular simulations

**DOI:** 10.1038/srep38715

**Published:** 2016-12-08

**Authors:** Archana Bhardwaj, Yogeshwar Vikram Dhar, Mehar Hasan Asif, Sumit K Bag

**Affiliations:** 1 Academy of Scientific and Innovative Research (AcSIR), CSIR-NBRI Campus, Lucknow, India; 2 Council of Scientific and Industrial Research - National Botanical Research Institute (CSIR-NBRI), Rana Pratap Marg, Lucknow, 226001, India

## Abstract

Single Nucleotide Polymorphisms (SNPs), an important source of genetic variations, are often used in crop improvement programme. The present study represented comprehensive *In silico* analysis of nucleotide polymorphisms in wild (*Solanum habrochaites*) and cultivated (*Solanum lycopersicum)* species of tomato to explore the consequence of substitutions both at sequence and structure level. A total of 8978 SNPs having Ts/Tv (Transition/Transversion) ratio 1.75 were identified from the Expressed Sequence Tag (EST) and Next Generation Sequence (NGS) data of both the species available in public databases. Out of these, 1838 SNPs were non-synonymous and distributed in 988 protein coding genes. Among these, 23 genes containing 96 SNPs were involved in traits markedly different between the two species. Furthermore, there were 28 deleterious SNPs distributed in 27 genes and a few of these genes were involved in plant pathogen interaction and plant hormone pathways. Molecular docking and simulations of several selected proteins showed the effect of SNPs in terms of compactness, conformation and interaction ability. Observed SNPs exhibited various types of motif binding effects due to nucleotide changes. SNPs that provide the evidence of differential motif binding and interaction behaviour could be effectively used for the crop improvement program.

The Solanaceae are the highly valuable plant taxon in terms of vegetable crops as well as agricultural utility.Various members of Solanaceae have been used as a model plants such as tomato (*Solanum lycopersicum*) and pepper, mainly to study the fruit development^1^. The genus *Solanum* is composed of approximately 1500 tomato species, originated in Central and North America, and spread all over the world. Despite the availability of numerous wild tomato species, only *Solanum habrochaites* has been exploited extensively for domestication purposes. This species typically grows on the western slopes of central Ecuador to central Peru, making it extremely tailored to the particular soil type and climatic conditions^2^. Moreover, this species has many unusual properties including maintenance of green color and hardness during fruit ripening^3^. Various changes have been observed in its physiological processes such as respiration, photosynthesis and Reactive Oxygen Species (ROS) productivity, which leads to its survival to cold stress conditions^4^. Earlier transcriptome studies^5^ of both the species have reported many changes in plants transferred from warm to cold conditions. Most of the phenotypic variation that exists among both species are listed in Table 1. All these properties indicated the presence of the desirable set of genes that could be used for the domestication of cultivated tomato (*Solanum lycopersicum*) species^3^. Fleshy fruits undergo a ripening process that includes a series of changes in the fruit color, texture, aroma, flavour and thus making the fruit edible^6^. Therefore, rapid phenotypic divergence associated with domestication is coupled to particular genomic loci that can be explored at the genetic level. In order to grasp the advance network of climacteric fruit ripening, numerous studies have been carried out that recommended tomato as a model plant due to its moderately short life cycle^7^ and availability of developmental mutant^8^. It is notable that there is a necessity to upgrade point-by-point information of genes directing fruit ripening, defense response against plant pathogen and other cellular processes.

Single Nucleotide Polymorphisms (SNPs) have gained the center stage in the era of molecular genetics, because of its high abundance, considerable information content and improvement in sequencing technology^9^. In tomato, SNPs were identified by various techniques such as Expressed Sequence Tag (EST)^10^, oligonucleotide arrays, intron and amplicon sequencing of Conserved Orthologous Set (COS) of genes and genotyping arrays^11^. The accomplishment of tomato genome sequence^12^ represents the most important asset in genetic research for deciphering Next Generation Sequencing (NGS) based SNPs. An enhancement in the ESTs dataset (from 8261 to 26019 of *Solanum habrochaites* species), available at http://www.ncbi.nlm.nih.gov/ and high throughput sequence dataset under Sequence Read Archive (SRA) have provided an oppertunity for the polymorphism detection. Generally, non-synonymous SNPs or substitutions have the ability to alter the amino acid sequence of a protein in contrast with synonymous SNPs which do not alter amino acid sequence. These non-synonymous substitutions could result in a biological change in any individual. Therefore, considerable attention has been given to non-synonymous SNPs (alter amino acid) because sequence change leads to various functional impact in terms of protein stability, which hampers the interaction with other proteins^13^.

Differential motif binding has a direct correlation with the gene expression, suggesting the functional consequence of binding variation^14^,^15^. In human genetics, various studies have explored the allele-specific motif binding and their role in human diseases^16^,^17^. Hence it is also important to examine the allele-specific differential binding effect of non-synonymous SNPs i.e motif binds to a specific allele of polymorphism but not to others. This information could be helpful in the study of the gene expression and chromatin modifications. The present study has been carried out to get insight about the differential motif binding due to SNPs among cultivated and wild tomato species.

Protein coding genes have a tendency to develop either negative or positive evolutionary routes in nature^18^. Previous study^19^ compared transcriptomic level selection pattern between one cultivated and six wild species, and characterized the footprints of positive selection. However, negative selection causes deleterious impacts on the host fitness and evolves at a slower rate in contrast to positive selection. Thus, we have given more emphasis on prediction of both positive and negative evolutionary selection pressure of genes to provide the detail picture of evolutionary selection pressure in studied species.

In this study, we focused on analysis of functional consequences of variations, by the consolidated utilization of ESTs (26019 and 298306 ESTs of *Solanum habrochaites* and *Solanum lycopersicum)* and high-throughput dataset under SRA (SRP041563) which enabled numerous SNPs mining among these two species. In addition, in-depth computational analysis of SNPs was performed at the sequence and structural level. Structural analysis enabled molecular dynamics simulations revealing many additional effects of SNPs in comparison to its native-type protein. The results may have the potential to improve our understanding of the consequence of SNPs in a novel way. Identified SNPs could be used as markers for traits of interest such as fruit ripening, color and texture under breeding program.

## Results

### ESTs and NGS-based SNP Discovery and chromosomal distribution

**Figure 1:**
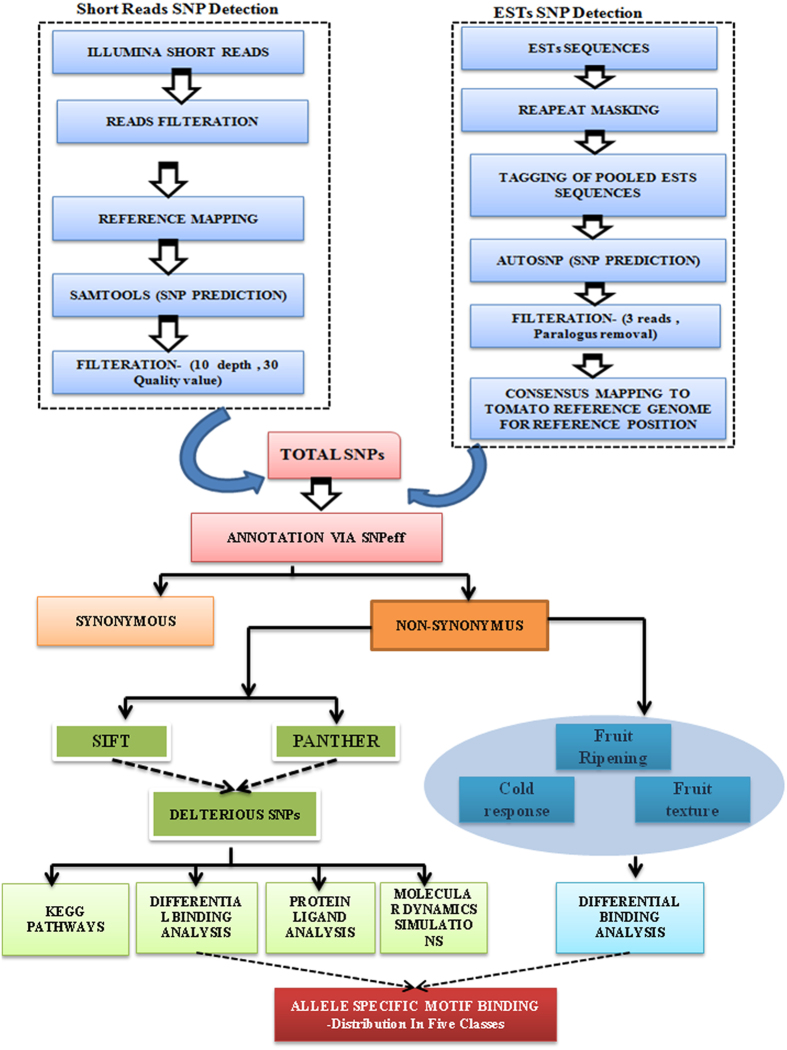
Flow chart representing the step wise procedure of NGS and ESTs SNP prediction and downstream analysis. ESTs and NGS illumina short reads are evaluated separately via *In silico* as illustrated via Short Reads SNPs Detection and ESTs SNPs detection stepwise procedure. Resulted cumulative set of all the SNPs are processed for the annotation, deleterious SNP prediction and analysed at sequence (differential motif binding, KEGG pathways) and structural level (Protein ligand, Molecular simulations).

**Figure 2:**
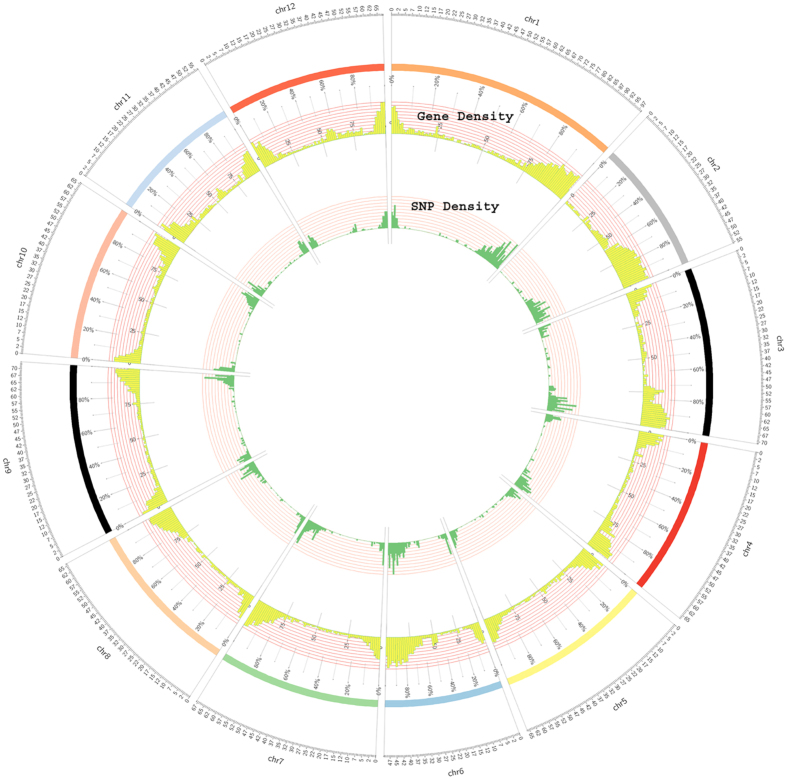
Circular graph showing SNP density along with Gene density. Graphical distribution of frequency of SNPs (green colour) along with gene density (yellow colour) between Heinz1706 and *Solanum habrochaites* (chromosome 1 to 12) under window size 1000/Kb.

A total of 8978 numbers of SNPs were predicted by comparing both the species as described in Fig. 1. The SNPs density (inner circle highlighted with green color) along with gene density (outer circle highlighted with yellow color) in the non-overlapping window of 1000/kb was assessed for each chromosome and plotted by Circos^20^ (Fig. 2). It clearly demonstrated a non-uniform pattern of SNPs distribution in all the chromosomes. A closer look of the data indicated that the density of both SNPs and genes were higher at one end of all the chromosomes except for chromosome 2 which showed SNPs hump from 34 Mb genomic regions (Fig. 2). Next, SNPs were classified on the basis of their nucleotide substitutions, either transition (purine-purine and pyrimidine- pyrimidine) or transversion (purine-pyrimidine and pyrimidine-purine). The rate of transition was 1.75 times higher than the transversion. The observed proportion of C/A+ T/G transversion was slightly lower (1601) than the C/G +A/T transversions (1662). Likewise, the transition to transversion ratio (Ts/Tv) was significantly (p < 0.001) higher at third codon position (2.01) than first (1.9) and second codon (1.89) positions. Observed transition to transversion ratio reflected the trend of genomic conservation at codon sites during evolution.

### Functional classification of SNPs

**Figure 3:**
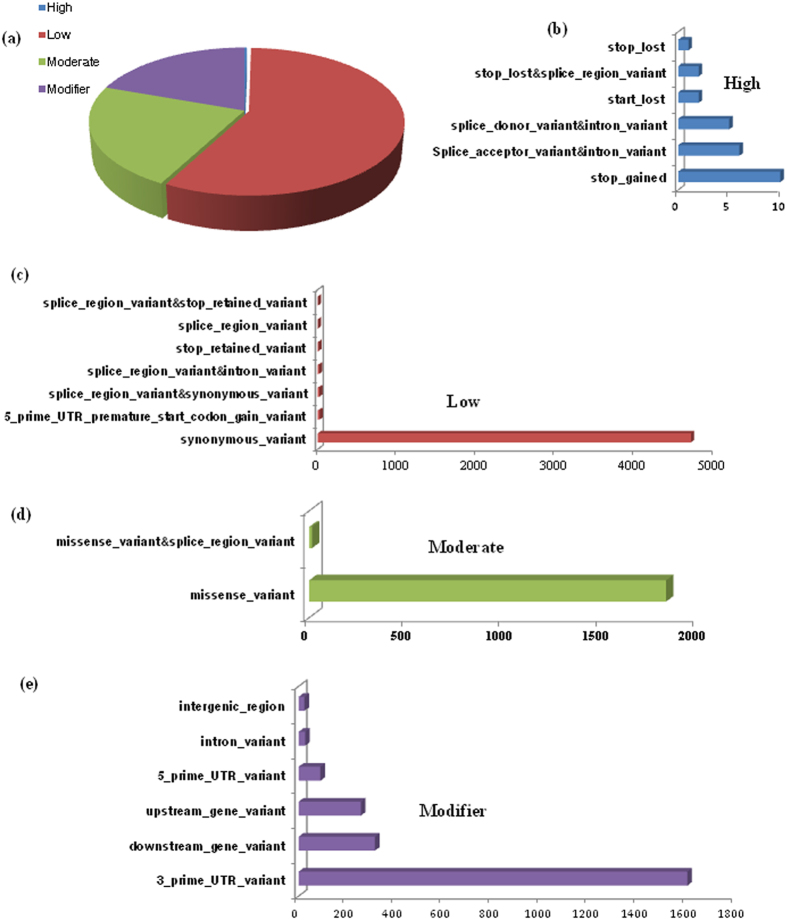
Multiple types of SNPs effects and their distribution. (**a**) Percentage contribution of SNPs in High, Low, Moderate and Modifier type of effects. Detail sub-classification of (**b**) High (**c**) Low (**d**) Moderate and (**e**) modifier groups.

All SNPs identified through the use of NGS and ESTs approach, were classified into various groups. Approximately 73% SNPs were found in coding region comprises both of synonymous (4739) and non-synonymous (1838) SNPs (Table 2), and distributed among 1840 and 988 protein coding genes, respectively. Using the SnpEff^21^ tool, we classified the overall impact of all the SNPs into four categories: low impact (53%), modifier (26%), moderate (20%), and high impact (1%) (Fig. 3a). High impact SNPs had the direct effect on the gene functionality, i.e caused either stop codon gain or stop codon loss in their respective genes. Stop gain and stop loss may lead to high level of functional consequences due to protein truncation and degradation of the transcript. Interestingly, we observed stop gain (G/A SNP) and stop lost (C/G SNP) in calmodulin 5/6/7/8-like and Glucan endo-1 3-beta-glucosidase 1 protein respectively, that play a role in the network of fruit ripening processes (Fig. 3b). Low impact SNPs consisted of synonymous SNPs whose amino acid remains unaltered in genes, therefore had a low impact on gene functionality. Gene observed under the effect of this group was Auxin response factor 8 (A/G SNP) which regulates the plant hormone regulatory pathway (Fig. 3c). Moderate impact was observed in non-synonymous SNPs which acquire the change in the amino acid due to nucleotide substitutions. This moderate type of impact was found in the HMG1 (high mobility group 1) gene known to involved in DNA regulatory processes (Fig. 3d). Modifier SNPs changed the functionality of respective gene and consist of both UTR (carries the binding site for various miRNA, transcription factors) and intergenic SNPs (Fig. 3e). As a result, transketolase 1 (T/A SNP) protein and Diphosphate-fructose-6-phosphate 1-phosphotransferase (A/G SNP) enzyme were found to consist of 3′ UTR SNPs.

### Evolutionary selection pressure under the effect of SNPs

**Figure 4:**
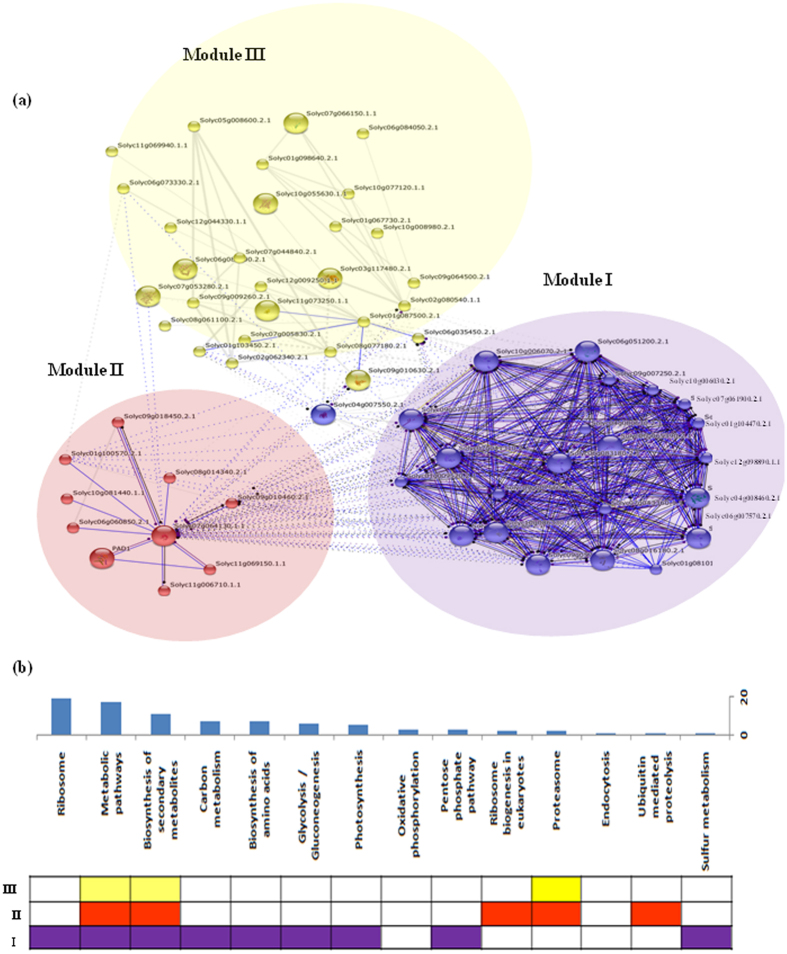
Network analysis of genes (purified and diversified groups) under the effect selection pressure and their enriched KEGG pathways. (**a**) String Interaction network showing the association of multiple genes and clustered into three distinct module via K mean clustering (**b**) Common and unique pathways among the three Modules.

The non-synonymous and synonymous substitution rate (Ka/Ks) were assessed to deduce the direction of natural selection over studied species. This analysis revealed the list of 205 and 34 genes representing the purified (Ka/Ks < 1.0) and diversified (Ka/Ks > 1), groups, respectively (Supplementary Table S1). The Ka/Ks of identified genes ranged from 0.02 to 0.3 in purified group while 1.0 to 4.16 in diversified group. Interactome network analysis provided the relationship of these genes and arranged into three major modules by the use of K-mean clustering as shown in Fig. 4a. Module I represented the densely packaged interaction among genes in comparison to the rest of the modules (II and III). Pathway analysis of depicted network indicated the enrichment of ribosome specific regulatory genes (Fig. 4b). Furthermore, common and unique pathways were analysed in order to get specific feature of each module. Counting the common pathways between the three modules, we observed that metabolic pathways and biosynthesis of secondary metabolism pathways were present in all the three modules. Whereas, glyconeogenesis and photosynthesis pathways were uniquely present in Module I (Fig. 4b purple Bar) and Ribosome biogenesis and ubiquitin mediated proteolysis pathways were uniquely present in Module II. Proteosome pathway was enriched in two modules (II and III) only. These results indicated the evolutionary selection pressure of genes regulating the metabolic pathways.

### Distinction of tolerable SNPs from Deleterious SNPs

In order to add another layer of refinement in SNP analysis, SIFT (Sorting Intolerant from Tolerant)^22^ and PANTHER (Protein ANalysis THrough Evolutionary Relationships)^23^ were used to distinguish the tolerable SNPs from deleterious SNPs. As a result, PANTHER predicted 110 SNPs to be deleterious, possessing the subPSEC score ≤−3 (5% of aggregate non-synonymous SNPs). SIFT predicted 277 SNPs possessing tolerance index score of ≤0.05, named as deleterious SNPs. Afterward, 28 non-synonymous SNPs were commonly depicted as deleterious under SIFT and PANTHER (Table 3). Next, 8 deleterious non-synonymous SNPs were found to be the part of the active site of eight individual genes. Complete functional details along with active site information are listed in Supplementary Table S2. Two deleterious SNPs replaced polar AA (amino acid) with hydrophobic AA (Ser67Ala, Ser170Phe). Overall consequence pinpoints that such type of SNPs are considered to be lethal and alter the critical component of biological reaction such as transcription process and DNA binding ability.

### Dynamic change in motif binding in allele-specific manner

**Figure 5:**
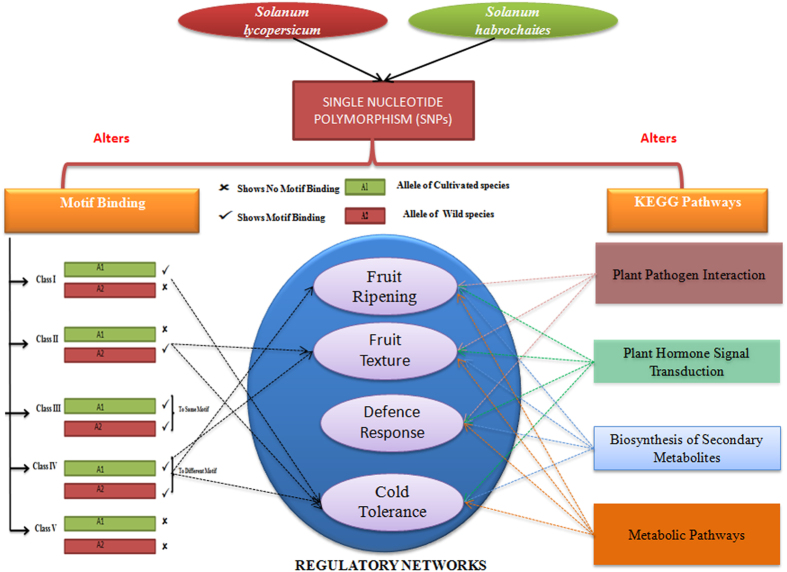
SNPs involved in multiple KEGG pathways and consist of differential motif binding event. Consequence of SNPs in genes regulating the fruit ripening, texture, cold and defense responses depicted via alteration in Motif binding behaviour and KEGG pathways.

Nucleotide change from cultivated to wild species resulted in preferential motif binding on either allele at sequence level, therefore called as allele-specific motif binding event. For better understanding, we created five possible combinations or classes as shown in Supplementary Fig. S1a. Further, we observed that Class I (3) and II (8) SNPs caused depletion in motif binding in wild and cultivated species respectively. Class III (4) SNPs exhibited no effect on motif binding due to allele variation, i.e both alleles show binding to the same motif, while class IV (6) SNPs showed dynamic change in motif binding from cultivated to wild species. No motif binding was observed in the rest of the deleterious SNPs (class V) as represented in Supplementary Fig. S1b. In this way, Class I, II and IV specific SNPs might be useful for the breeding program. Besides this, a collective list of genes regulating the complex network of fruits ripening and other complexes was prepared, which allowed us to investigate the consequence of predicted SNPs in these complexes. As a result, 96 SNPs were found to be the part of the gene regulating traits such as fruit ripening, cold response, trichome development and fruit texture. This study revealed that one of the texture regulatory gene Fasciclin-like arabinogalactan protein 13 carries A/G allelic variation, where allele A binds to Myb motif while allele G binds to Storekeeper motif, indicating the dynamic change in motif binding, therefore included in Class IV. A similar dynamic change in motif binding (from Myb to Homeodomain) was found in beta-glucosidase D4 gene carrying A/T point variation represents the Class IV motif binding effect as listed in Supplementary Table S3. Overall the result indicated that allelic effect had a wider biological role in the multiple fruit ripening complexes as shown in the Fig. 5.

### Structural modelling and consequence of deleterious SNPs depicted via protein-ligand complexes

To directly readout the effect of deleterious SNPs, we superimposed the native (represent the allele of cultivated species) and mutant (represent the allele of wild species) protein models after the energy minimization. Six SNPs significantly changed the structure alignment of native and mutant protein models (Supplementary Fig. S2a–f). Detailed analysis presented the immense deviation between the native and Tyr416Asp mutant structures of HMG1 protein pointing towards alteration in the structural geometry (Supplementary Fig. S2a). Superimposed structure of native and Ser67Ala mutant of H1 Histone-like protein showed a change in loop to helix conformation after energy minimization of protein structures (Supplementary Fig. S2b). This change may affect the functionality of Histone-like protein which could lead to the change in the DNA binding pattern with their interacting members. Other three deleterious SNPs (Val133Ala, Ser170Phe, Lys67Asn), although observed to be forming the loop conformation in both native and mutant models (Histone H1, Ribonuclease P protein and Reticulon family protein), changed the 3D conformation in mutant as shown in Supplementary Fig. S2c–e. One of the SNP (Leu164Pro) in elongation factor G gene, brought the change from loop (native) to sheet (mutant) conformation in 3-dimensional protein structures (Supplementary Fig. S2f). This change illustrated the higher stability of the mutant structure of elongation factor G protein as compared to its native form.

**Figure 6:**
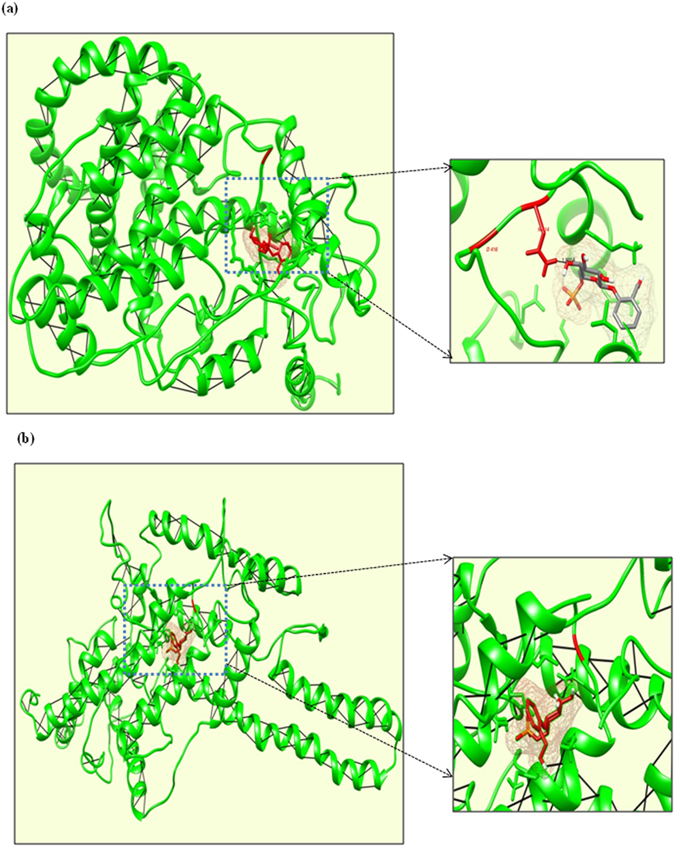
Interaction of p53 ligand with native and mutant models of HMG1 protein. (**a**) p53 Ligand binds with native HMG1 protein close to SNP site (**b**) p53 ligand binds with mutant HMG1 protein far away from SNP site. Green ribbons representing the 3D conformation of protein models and red indicating the protein ligand. Arrow demarcating the zoom region of ligand targeted site (highlighted with red color).

To analyse interaction behaviour using docking simulations, one of the protein HMG1 consist of Tyr416Asp AA change, was selected. This SNP affected the active site of the HMG1 protein which could be clearly implied through docking simulations. HMG1 is known for its interaction with p53 ligand. Both the native HMG1-p53 (Fig. 6a) and Tyr416Asp mutant HMG1-p53 (Fig. 6b) complexes revealed similar interaction pattern with an almost similar energy value of −273.8 kcal/mol and −267.4 kcal/mol respectively, with single H-bond. A closer look at the interaction data indicated that in the native HMG1-P53 complex, the ligand fits in the proper pocket and binds with the active site residues at preferred position of N414 (shown via arrow to zoom in). While in the Tyr416Asp mutant HMG1-p53 complex, substitution at the 416 position had changed not only the conformation, but also the pose and site of binding too. Here, ligand binds far away positioned amino acid from the SNP site due to the change in conformation of proteins. This result indicated that modification had affected the interaction ability of HMG proteins due to conformation change brought by deleterious SNP.

### Structural deviation and fluctuation due to deleterious SNPs

**Figure 7:**
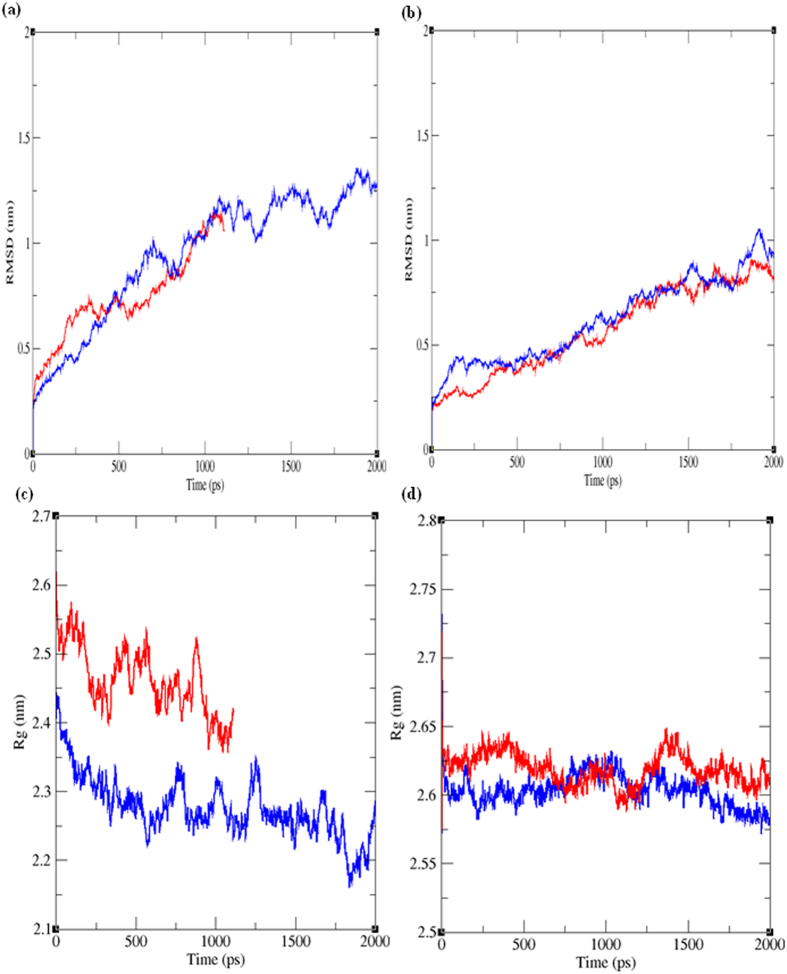
Molecular simulations of various Native and Mutant protein models carrying deleterious SNPs. (**a**) RMSD of native and Cys284Arg mutant HMG1 proteins models (**b**) RMSD of native and Asp115Glu mutant PR1 protein models (**c**) Radius of gyration of native and Cys284Arg mutant HMG1 protein models (**d**) Radius of gyration of native and Asp115Glu mutant PR1 protein models. All the simulations were performed at 2000 picoseconds (ps). Blue line represents the native and red line indicates associated mutant proteins.

For the detailed functionality of genes carrying the 28 deleterious SNPs, pathway analysis was performed using KOBAS web server. Deleterious SNPs were predicted to be the part of base excision repair pathways (affected the HMG1-high mobility group protein B gene), plant hormone signal transduction (affected the T1R1 and PR1 b- pathogen responsive 1b) and plant pathogen interaction pathway (affected the PR1-pathogen responsive 1) (Supplementary Fig. S3a–d). Under the molecular simulations, we observed that complex of native PR-1 protein (blue) shows almost same pattern of Root Mean Square Deviation (RMSD) growth with respect of time, with slight peaks between 0 to 500 ps and 1750 to 2000 ps (Fig. 7a). After a certain time range, Cys284Arg mutant PR-1 protein (red) obtained convergence towards its folding as compared to the native structural fold, which was an indication of change in required functional distance between the atoms, thus change in functional geometry. A clear change in the peaks of RMSD was detected in Asp115Glu mutant HMGB1 protein, almost following the peak & valley pattern of the native protein up to 1 ns (nanosecond) and revealed interrupted peak line at 1100 ns, while the native one maintains a pattern in remaining time intervals (Fig. 7b). It further indicated that change in functional geometry of protein was affected specifically on the basis of scalar distance between the atom sets of corresponding amino acid.

Radius of gyration (Rg) represents the measure of compactness of the protein conformation in biomolecular simulations. In one hand, the native and Cys284Arg mutant PR-1 protein showed a clear difference in gyration radius where native protein exhibited affinity towards convergence and maintains the steady flow between 2.4 to 2.2 nm (nanometer). Whereas the mutant protein represented its compactness point at 1100 ps between 2.6 to 2.4 nm (Fig. 7c). It indicated the effect of mutation over the structural compactness and thus effect on the folding of PR-1 protein. On the other hand, the native and Asp115Glu mutant HMGBI protein, showed almost similar pattern of gyration plot with overlap of peaks at 750 ps to 1250 ps range (Fig. 7d). Following up the similar pattern of gyration radius and overlap for a time range show that mutation was also favourable for maintaining the compactness of structure.

## Discussion

In plants, the majority of traits of interest are linked with SNPs and are thought to bring the individual variation, community diversity and the evolution of species. The link between single nucleotide change and gene function has been reported for a number of traits^24^. Modern science is mainly focused on finding the differential behaviour in genomes by utilizing microarray, transcriptome (in the form of up and down regulated genes) and ChIP-seq (in the form of differential peaks) like high throughput approaches. The present study was centered around finding the differential behaviour of non-synonymous SNPs in terms of their motif binding and put forward a methodological approach for SNPs analysis. In this study, we applied more stringent filters for SNP detection as compared to the previous report^25^. Numerous SNPs were identified by making the use of ESTs and sequencing approach as discussed in preceding studies^26^. Identified SNPs were found to be under the influence of transition biases i.e occurrence of higher transition as compared to transversion^27^. However, a contrasting result was observed in ginger EST leaves sequences^28^. The occurrence of higher transition as compared to transversion might be associated to the high frequency of the C to T variation after methylation^29^. The frequency of SNPs was highest at third and lowest at second codon positions^30^. Furthermore, predicted SNPs density was found to be in accordance with gene density, over each chromosome. A recent report^31^ claimed the major divergence at heterochromatin as compared to euchromatin regions in their studied species. Here, we found that chromosome 1 consists of maximum variation as expected due to its largest size, which was contradictory from earlier study^11^. Valuable resource of functional data was depicted in the form of SNPs identified in UTR, coding and splice site regions that could provide the ample support for association studies.

The stringency of functional or structural constraints is thought to be a noteworthy key component behind the rate of amino acid substitutions^32^. We identified 205 purifying genes (Ka/Ks < 1), which tend to evolve slower than proteins with weaker constraints and 34 diversifying genes (Ka/Ks > 1) that are known to evolve quickly and tend to acquire a new function. Different threshold were applied in the other reports for positive (purifying) and negative (diversifying) groups such as in pacific white shrimp^18^, authors adopted Ka/Ks > 0.6 and Ka/Ks < 0.2 to be the realistic parameter for the diversifying and purifying respectively, in *Eucalyptus grandis*^33^ Ka/Ks < 0.15 for positive and Ka/Ks > 0.5 for negative selection. Many purifying genes were traced among cultivated and wild tomato species^34^ and provides the conclusive influence of SNPs in the evolutionary study.

SNPs are known to be associated with many aspects of human development and diseases. In plants, SNPs are reported to be regulating various Quantitative Trait Loci (QTL) responsible for cold and disease resistance such as such as blight, bacterial canker and gray mold^35^,^36^. In this study, various SNPs were observed on chromosome 5 and 11, already reported for their involvement in QTLs linked with reduced self-seed, late blight disease which influences fruit size, canopy density and plant size^37^,^38^ and appears to be convincing evidence for the significance of the other predicted SNPs. Differential binding had elucidated the significance of each allele in terms of their motif binding ability. The comparable conclusion comes from another study^39^ where researchers observed the influence of SNPs on WRKY domain and found a lesser TGAC binding affinity in WKRY domain due to the presence of non-synonymous SNP. Interpretation of non-synonymous SNPs carrying genes which are regulating fruit ripening, texture and cold responses along with their differential behaviour could provide in-depth information to the biologist.

The pathways of biosynthesis of secondary metabolites and plant hormone signal transduction are known for their role in fruit ripening and defense responses^40^. It has been reported that changes in cell wall structure and the conversion of starch into simple sugars play a significant contribution during fruit ripening^41^. Deleterious SNPs were found to be the part of such biological pathways (plant hormone signal transduction and metabolic pathways) and thus enhance the significance of the present study. Metabolites act as a key component of tomato fruit flavour and participate in plant defense responses against pathogens and herbivore^42^. Involvement of non-synonymous SNPs in various metabolic pathways (glycine, serine and threonine metabolism, carbon metabolism and biosynthesis of secondary metabolites) directly implied their role in variation among studied species. Furthermore, 11 SNPs were identified in carotenoid genes, known for its role in fruit ripening and impart colors of fruits which could be used to understand the fruit colouring mechanism. Involvement of 58 SNPs in genes reported to be playing a role in the fruit texture, could be addressed in detail at breeding level. Also, 26 SNPs were identified in the cold-responsive genes which could be used to understand the complex mechanisms of cold stress because cultivated species are unable to tolerate freezing. RMSD and Rg values were considered as selective attributes via molecular simulation analysis. Molecular simulation analysis revealed the SNPs influence over the proteins playing a role in the defense response (PR1) and metabolic activities; both are noteworthy phenotypic difference known to exist in both the species. A similar approach was applied in earlier studies^43^,^44^,^45^ to study the influence of non-synonymous SNPs. Present study must enhance the knowledge of the scientific community and provide support for the future breeding program.

## Materials and Methods

### Pre-processing of Transcriptome sequencing data and ESTs sequences

Transcriptome sequencing data (SRP041563) was downloaded from the SRA (http://www.ncbi.nlm.nih.gov/sra/) belonging to proximal and distal leaves of tomato of *Solanum lycopersicum* and *Solanum habrochiates*. We used SRA toolkit to get the single end reads (42 samples) in fastq format. In parallel, the EST sequence data of *Solanum lycopersicum* and *Solanum habrochaites* comprising a large amount of leaf, fruit, Trichome I and Trichome IV specific cDNA sequences, were downloaded from National Center for Biotechnology Information (NCBI) in fasta format. As ESTs were sequenced only once, sometimes the data has low-quality sequence and repeats generate problems during downstream analysis. To remove the interspersed repeats and low complexity regions, RepeatMasker^46^ was run on EST datasets of both species separately against plant based Repbase libraries (http://www.girinst.org/server/RepBase/index.php). This reduced the chance of biases in SNP analysis and the overall noise in EST sequences.

### SNP analysis in Illumina short reads and ESTs sequences

Illumina short reads of wild and cultivated species were mapped to the reference genome of *Solanum lycopersicum* 2.5 version utilizing the Tophat v2.0^47^ with the default option of -n/mismatch 2, -i/min-intron-length 50 except for -p/num-threads that set to 8. Only reliable mapped reads were considered for SNP calling and unmapped reads were discarded. SNP positions within mapped reads were predicted by use of samtools^48^. VCFtools^49^ was employed on the raw Variant Calling Format (VCF) files for the minimum depth (DP) 10 and SNP quality (Q) 30 to get high-quality SNPs as described in Fig. 1 under short reads SNP detection workflow. For ESTs based SNPs analysis, AutoSNP v 2^50^ tool was used with some modifications, and SNPs were differentiated from sequencing error according to sequence redundancy. Pre-processed EST sequences were assembled using the Contig Assembly Program v.3 (CAP3) program^51^ within the AutoSNP pipeline at 85% identity (-p) and 100 bp overlap (-o). The resulted ACE format files were then parsed for SNP identification using custom scripts. Few filtration steps were applied in ESTs dataset, in order to diminish false SNP prediction caused by sequencing errors such as - depth of reads (minimum 3/3 reads belongs to both species) and removal of paralogous polymorphism at SNP site as shown in Fig. 1 under ESTs SNP detection workflow. Rest of indels and variants involving more than one nucleotide change in both (short read as well as EST) approaches were excluded. Furthermore, we performed the blast of the ESTs contig at e-value ≤ 1e-10 and 90% identity, against the tomato genic sequences to get their position in reference genome. A portion of the SNPs comprised of allelic variation with respect to (w.r.t) genic sequences. In this manner, such SNPs were excluded in further processing.

### SNPs classification and interactome network of purified genes during selection pressure of SNPs

To annotate the effect of SNPs (synonymous and non-synonymous), SnpEff^21^ software was used. Identified SNPs were scanned for the ratio of the number of non-synonymous substitutions per non-synonymous site (K_a_), to the number of synonymous substitutions per synonymous site (K_s_), in the given timeframe. In this way, cultivated and wild allele carrying gene sequences were created based on the SNP position and compared in Ka/Ks_calculator^52^ tool with an optional parameter of standard genetic code and NG method. Two variables (positive and negative) were created by Ka/Ks ratio, i.e genes with Ka/Ks ≥ 1 falls in the diversifying group while genes with Ka/Ks ≤ 1 genes fall in the purifying group. These two groups were further analysed for their interaction network in STRING database^53^ (http://string-db.org/).

### Assessment of deleterious SNPs under PANTHER and SIFT and their active site prediction

To find out the potential functional effect of amino acid substitution on corresponding proteins, SIFT^22^ (subPSEC score ≤−3) and PANTHER^23^ (tolerance index score of ≤0.05) were used for the detection of deleterious SNPs. Three dimensional structure of both native and mutant proteins were produced by the Phyre2 server^54^ available at www.sbg.bio.ic.ac.uk/phyre2/html/ under intensive mode. Simultaneously, active sites of protein model carrying deleterious SNP were predicted by using CASTP web server (http://sts.bioe.uic.edu/castp/). Furthermore, native and mutant structures were superimposed in UCSF-Chimera molecule viewer tool after energy minimization using Needleman-Wunch alignment method, applying gap extension penalty 1, gap opening penalty 12 and structure score > 30% with distance range of 2.0 A.

### Probable consequence of deleterious SNPs at sequence level

To understand SNP impact at the sequence level, differential binding events were predicted by utilizing CIS-BP web server (http://cisbp.ccbr.utoronto.ca/TFTools.php), where sequences of each of 10 bp (in upstream and downstream) from SNPs site were extracted and used as input in CIS-BP web server^55^ by the use of 8mer motif model. Moreover, SNPs were classified into five classes based on motif binding ability of each SNP allele as demonstrated in Supplementary Fig. S1a.

### Protein conformation changes at structural level

One of the deleterious SNP was randomly selected for protein-ligand analysis. Docking analysis was performed to predict the putative modification of binding modes of studied ligand (p53) with selected protein (HMG1) using AutoDock 4.0 (http://autodock.scripps.edu). The grid size was set to cover acting domains present in selected protein with the grid spacing of 0.375 Å. Genetic algorithm (GA) was applied as searching parameter with 10 numbers of GA runs and setting population size 150, a maximum number of energy evaluations was set to 25,00,000 with considering the maximum number of generations to 27,000. The lowest binding energy conformation with H-Bonds in cluster was considered as the most favourable docking pose. Protein-ligand complexes obtained from AUTODOCK 4 were further viewed in UCSF-Chimera molecule viewer tool for better analysis of interaction. In each case, 10 different docking arrangements were produced. The conformations obtained as a result of rigid body docking were sorted by total binding energy, hydrogen bonds formed, bond lengths and close contacts between enzyme active sites.

### Detail Annotation and molecular simulation of deleterious SNPs at structural level

To elucidate key role of deleterious SNPs at a biological level, Kobas web server (http://kobas.cbi.pku.edu.cn/home.do) was used for pathway analysis. Furthermore, molecular simulations were performed on the selective 2 non-synonymous SNPs to gain insight into the impact of deleterious SNPs in terms of protein functionality. All the molecular simulations were carried out using Gromacs program^56^ opting GROMOS96 54a7 force field and SPCE water model. A cubic box with 1.0 nm spacing was generated for each protein, followed by the energy minimization using the steepest descent method. After minimization, equilibrium (canonical NVT ensemble) step was initiated using leap-frog integrator and set the LINCs order value 4. Again, Leap-frog integrator was used for the production MD run, considering all bond constrains with PME for long-range electrostatics.

## Additional Information

**How to cite this article**: Bhardwaj, A. *et al*. *In Silico* identification of SNP diversity in cultivated and wild tomato species: insight from molecular simulations. *Sci. Rep.* **6**, 38715; doi: 10.1038/srep38715 (2016).

**Publisher's note:** Springer Nature remains neutral with regard to jurisdictional claims in published maps and institutional affiliations.

## Supplementary Material

Supplementary Figures and Tables

## Figures and Tables

**Table 1:** Phenotypic features of Solanum lycopersicum and Solanum habrochaites.

S. NO	Character	S. lycopersicum	S. habrochaites
1	Type	Cultivated	Wild
2	Fruit Colour	Red (0.43–0.93 gram per fruit)	Green hairy (1–2 gram per fruit)
3	Viral Resistance	Sensitive	Resistant
4	Bacterial Resistance	Sensitive	Resistant
5	Glandular trichomes	Contain terpens in large amount	Contain methylketones in large amount
6	Cold Tolerance	Highly Sensitive	Highly tolerable
7	Nutritional Content	High content of Zn, low content of Na, K, Ca, Fe	High content of Na, K, Ca, Fe and Mn
8	Salt stress tolerance	Sensitive	Tolerant
9	Ethylene content	High during ripening	Unsual pattern of ethylene

**Table 2:** Distribution of SNPs.

Type of region	Type of effect	Frequency of SNPs
**CODING**	SYNONYMUS	4739
	NON-SYNONYMUS	1838
	DELETERIOUS SNPS	28
	ALLELE- SPECIFIC BINDING	17
**UTR**		1695
**PROMOTER**		572
**SPLICE REGION**		54
**others (Intergenic/Inton)**		80

**Table 3:** Deleterious SNPs score predicted in SIFT and Panther.

Locus ID	AA change	SIFT -SCORE	PANTHER -SCORE
Solyc06g036050	Leu42Pro	0	−5.5711
Solyc08g082070	Tyr416Asp	0.02	−5.39792
Solyc01g090690	Leu164Pro	0	−5.11611
Solyc05g006730	Asp105Asn	0	−4.67438
Solyc06g083270	Cys254Arg	0	−4.21414
Solyc08g006330	Ile343Met	0.03	−4.15168
Solyc01g099410	Leu103Met	0.01	−4.01464
Solyc04g011400	Gly315Arg	0.02	−4.0083
Solyc04g081850	Gly155Ala	0	−3.82874
Solyc04g081880	Ser170Phe	0	−3.8245
Solyc09g074520	Cys284Arg	0.01	−3.70012
Solyc08g005680	Glu140Gly	0.03	−3.54009
Solyc01g111450	Glu91Gly	0.01	−3.4529
Solyc05g018810	Lys71Asn	0	−3.39811
Solyc05g056390	Asn32Lys	0.04	−3.36092
Solyc02g091280	Ile120Phe	0.04	−3.22634
Solyc02g080810	Leu250Phe	0.02	−3.21987
Solyc09g011140	Asn150Tyr	0.04	−3.19296
Solyc07g014700	Leu588Pro	0.02	−3.1897
Solyc09g066100	Val133Ala	0	−3.18709
Solyc04g074980	Ser270Cys	0.02	−3.1504
Solyc05g056000	Leu302Pro	0	−3.10015
Solyc05g056000	Lys289Arg	0.05	−3.06407
Solyc09g007020	Ser145Cys	0.02	−3.05517
Solyc00g174340	Ser144Cys	0.02	−3.0551
Solyc02g082700	Asp115Glu	0	−3.0519
Solyc02g084240	Ser67Ala	0	−3.04107
Solyc06g036430	Lys67Asn	0	−3.01878

## References

[b1] Kimura S. & Sinha N. Tomato (Solanum lycopersicum): A Model Fruit-Bearing Crop. CSH protocols 2008, pdb emo105, doi: 10.1101/pdb.emo105 (2008). 21356708

[b2] Momotaz A., Scott J. W. & Schuster D. J. Solanum habrochaites accession LA1777 recombinant inbred lines are not resistant to tomato yellow leaf curl virus or tomato mottle virus. HortScience 42, 1149–1152 (2007).

[b3] Dal Cin V., Kevany B., Fei Z. & Klee H. J. Identification of Solanum habrochaites loci that quantitatively influence tomato fruit ripening-associated ethylene emissions. Theoretical and applied genetics 119, 1183–1192 (2009). 19680624 10.1007/s00122-009-1119-x

[b4] Paupiere M. J., van Heusden A. W. & Bovy A. G. The metabolic basis of pollen thermo-tolerance: perspectives for breeding. Metabolites 4, 889–920 (2014). 25271355 10.3390/metabo4040889 PMC4279151

[b5] Chen H. . A comparison of the low temperature transcriptomes of two tomato genotypes that differ in freezing tolerance: Solanum lycopersicum and Solanum habrochaites. BMC Plant Biol 15, 132, doi: 10.1186/s12870-015-0521-6 (2015). 26048292 PMC4458020

[b6] Manning K. . A naturally occurring epigenetic mutation in a gene encoding an SBP-box transcription factor inhibits tomato fruit ripening. Nature genetics 38, 948–952 (2006). 16832354 10.1038/ng1841

[b7] Pino L. E. . The Rg1 allele as a valuable tool for genetic transformation of the tomato ‘Micro-Tom’ model system. Plant methods 6, 23, doi: 10.1186/1746-4811-6-23 (2010). 20929550 PMC2958934

[b8] Thompson A. J. . Molecular and genetic characterization of a novel pleiotropic tomato-ripening mutant. Plant physiology 120, 383–390 (1999). 10364389 10.1104/pp.120.2.383 PMC59276

[b9] Mammadov J., Aggarwal R., Buyyarapu R. & Kumpatla S. SNP markers and their impact on plant breeding. Int J Plant Genomics 2012, 728398, doi: 10.1155/2012/728398 (2012). 23316221 PMC3536327

[b10] Jimenez-Gomez J. M. & Maloof J. N. Sequence diversity in three tomato species: SNPs, markers, and molecular evolution. BMC Plant Biol 9, 85, doi: 10.1186/1471-2229-9-85 (2009). 19575805 PMC3224693

[b11] Sim S. C. . High-density SNP genotyping of tomato (Solanum lycopersicum L.) reveals patterns of genetic variation due to breeding. PLoS One 7, e45520, doi: 10.1371/journal.pone.0045520 (2012). 23029069 PMC3447764

[b12] Tomato Genome C. The tomato genome sequence provides insights into fleshy fruit evolution. Nature 485, 635–641 (2012). 22660326 10.1038/nature11119 PMC3378239

[b13] Yates C. M. & Sternberg M. J. Proteins and domains vary in their tolerance of non-synonymous single nucleotide polymorphisms (nsSNPs). Journal of molecular biology 425, 1274–1286 (2013). 23357174 10.1016/j.jmb.2013.01.026

[b14] Kasowski M. . Variation in transcription factor binding among humans. Science 328, 232–235 (2010). 20299548 10.1126/science.1183621 PMC2938768

[b15] Reddy T. E. . Effects of sequence variation on differential allelic transcription factor occupancy and gene expression. Genome research 22, 860–869 (2012). 22300769 10.1101/gr.131201.111 PMC3337432

[b16] Ni Y., Hall A. W., Battenhouse A. & Iyer V. R. Simultaneous SNP identification and assessment of allele-specific bias from ChIP-seq data. BMC Genet 13, doi: 10.1186/1471-2156-13-46 (2012). PMC3434080 22950704

[b17] Cavalli M. . Allele-specific transcription factor binding to common and rare variants associated with disease and gene expression. Human genetics 135, 485–497 (2016). 26993500 10.1007/s00439-016-1654-x PMC4835527

[b18] Liu C., Wang X., Xiang J. & Li F. EST-derived SNP discovery and selective pressure analysis in Pacific white shrimp (Litopenaeus vannamei). Chinese Journal of Oceanology and Limnology 30, 713–723 (2012).

[b19] Koenig D. . Comparative transcriptomics reveals patterns of selection in domesticated and wild tomato. Proc Natl Acad Sci USA 110, E2655–2662, doi: 10.1073/pnas.1309606110 (2013). 23803858 PMC3710864

[b20] Krzywinski M. . Circos: an information aesthetic for comparative genomics. Genome Res 19, 1639–1645 (2009). 19541911 10.1101/gr.092759.109 PMC2752132

[b21] Cingolani P. . A program for annotating and predicting the effects of single nucleotide polymorphisms, SnpEff: SNPs in the genome of Drosophila melanogaster strain w1118; iso-2; iso-3. Fly 6, 80–92 (2012). 22728672 10.4161/fly.19695 PMC3679285

[b22] Ng P. C. & Henikoff S. SIFT: Predicting amino acid changes that affect protein function. Nucleic acids research 31, 3812–3814 (2003). 12824425 10.1093/nar/gkg509 PMC168916

[b23] Thomas P. D. . PANTHER: a library of protein families and subfamilies indexed by function. Genome research 13, 2129–2141 (2003). 12952881 10.1101/gr.772403 PMC403709

[b24] Shirasawa K. . Genome-wide association studies using single nucleotide polymorphism markers developed by re-sequencing of the genomes of cultivated tomato. DNA research 20, 593–603 (2013). 23903436 10.1093/dnares/dst033 PMC3859326

[b25] Barker G., Batley J., O’Sullivan H., Edwards K. J. & Edwards D. Redundancy based detection of sequence polymorphisms in expressed sequence tag data using autoSNP. Bioinformatics 19, 421–422 (2003). 12584131 10.1093/bioinformatics/btf881

[b26] Labate J. A. & Baldo A. M. Tomato SNP discovery by EST mining and resequencing. Molecular Breeding 16, 343–349 (2005).

[b27] Riju A., Chandrasekar A. & Arunachalam V. Mining for single nucleotide polymorphisms and insertions/deletions in expressed sequence tag libraries of oil palm. Bioinformation 2, 128–131 (2007). 21670789 10.6026/97320630002128 PMC2255072

[b28] Chandrasekar A., Riju A., Sithara K., Anoop S. & Eapen S. J. Identification of single nucleotide polymorphism in ginger using expressed sequence tags. Bioinformation 4, 119–122 (2009). 20198184 10.6026/97320630004119 PMC2828891

[b29] Zhao H. . The study of neighboring nucleotide composition and transition/transversion bias. Science in China Series C: Life Sciences 49, 395–402 (2006). 16989286 10.1007/s11427-006-2002-5

[b30] Castle J. C. SNPs occur in regions with less genomic sequence conservation. PLoS One 6, e20660, doi: 10.1371/journal.pone.0020660 (2011). 21674007 PMC3108954

[b31] Suresh B. V., Roy R., Sahu K., Misra G. & Chattopadhyay D. Tomato genomic resources database: an integrated repository of useful tomato genomic information for basic and applied research. PLoS One 9, e86387, doi: 10.1371/journal.pone.0086387 (2014). 24466070 PMC3897720

[b32] Kimura M. Preponderance of synonymous changes as evidence for the neutral theory of molecular evolution. Nature 267, 275–276 (1977). 865622 10.1038/267275a0

[b33] Novaes E. . High-throughput gene and SNP discovery in Eucalyptus grandis, an uncharacterized genome. BMC Genomics 9, 312, doi: 10.1186/1471-2164-9-312 (2008). 18590545 PMC2483731

[b34] Corrado G., Piffanelli P., Caramante M., Coppola M. & Rao R. SNP genotyping reveals genetic diversity between cultivated landraces and contemporary varieties of tomato. BMC Genomics 14, 835, doi: 10.1186/1471-2164-14-835 (2013). 24279304 PMC4046682

[b35] Coaker G. L. & Francis D. M. Mapping, genetic effects, and epistatic interaction of two bacterial canker resistance QTLs from Lycopersicon hirsutum. Theoretical and Applied Genetics 108, 1047–1055 (2004). 15067391 10.1007/s00122-003-1531-6

[b36] Zhang L. P., Lin G. Y., Nino-Liu D. & Foolad M. R. Mapping QTLs conferring early blight (Alternaria solani) resistance in a Lycopersicon esculentumÃ— L. hirsutum cross by selective genotyping. Molecular Breeding 12, 3–19 (2003).

[b37] Finkers R. . Three QTLs for Botrytis cinerea resistance in tomato. Theoretical and Applied Genetics 114, 585–593 (2007). 17136515 10.1007/s00122-006-0458-0

[b38] Haggard J. E., Johnson E. B. & Clair D. A. S. Linkage relationships among multiple QTL for horticultural traits and late blight (P. infestans) resistance on chromosome 5 introgressed from wild tomato Solanum habrochaites. G3: Genes| Genomes| Genetics 3, 2131–2146 (2014). 10.1534/g3.113.007195 PMC3852376 24122052

[b39] Parida S. K., Mukerji M., Singh A. K., Singh N. K. & Mohapatra T. SNPs in stress-responsive rice genes: validation, genotyping, functional relevance and population structure. BMC Genomics 13, 426, doi: 10.1186/1471-2164-13-426 (2012). 22921105 PMC3562522

[b40] Rai R. Role of Secondary Metabolites in Plant Defence Mechanism. Global Journal For Research Analysis 5, 82–84 (2016).

[b41] Seymour G. B., Chapman N. H., Chew B. L. & Rose J. K. Regulation of ripening and opportunities for control in tomato and other fruits. Plant Biotechnol J 11, 269–278 (2013). 22958755 10.1111/j.1467-7652.2012.00738.x

[b42] Tzin V. . Altered Levels of Aroma and Volatiles by Metabolic Engineering of Shikimate Pathway Genes in Tomato Fruits. AIMS Bioengineering 2, 75–92 (2015).

[b43] Rasal K. D., Shah T. M., Vaidya M., Jakhesara S. J. & Joshi C. G. Analysis of consequences of non-synonymous SNP in feed conversion ratio associated TGF-beta receptor type 3 gene in chicken. Meta Gene 4, 107–117 (2015). 25941634 10.1016/j.mgene.2015.03.006 PMC4412971

[b44] Hirakawa H. . Genome-wide SNP genotyping to infer the effects on gene functions in tomato. DNA research 20, 221–233 (2013). 23482505 10.1093/dnares/dst005 PMC3686429

[b45] Kamaraj B. & Purohit R. In silico screening and molecular dynamics simulation of disease-associated nsSNP in TYRP1 gene and its structural consequences in OCA3. Biomed Res Int 2013, 697051, doi: 10.1155/2013/697051 (2013). 23862152 PMC3703794

[b46] Tarailo-Graovac M. & Chen N. Using RepeatMasker to identify repetitive elements in genomic sequences. Current protocols in bioinformatics/editoral board, Andreas D. Baxevanis … [et al.] Chapter 4, Unit 4 10 (2009). 10.1002/0471250953.bi0410s25 19274634

[b47] Trapnell C., Pachter L. & Salzberg S. L. TopHat: discovering splice junctions with RNA-Seq. Bioinformatics 25, 1105–1111 (2009). 19289445 10.1093/bioinformatics/btp120 PMC2672628

[b48] Li H. . The sequence alignment/map format and SAMtools. Bioinformatics 25, 2078–2079 (2009). 19505943 10.1093/bioinformatics/btp352 PMC2723002

[b49] Handsaker R. E. . The variant call format and VCFtools. Bioinformatics 27, 2156–2158 (2011). 21653522 10.1093/bioinformatics/btr330 PMC3137218

[b50] Barker G., Batley J., O’Sullivan H., Edwards K. J. & Edwards D. Redundancy based detection of sequence polymorphisms in expressed sequence tag data using autoSNP. Bioinformatics 19, 421–422 (2003). 12584131 10.1093/bioinformatics/btf881

[b51] Huang X. & Madan A. CAP3: A DNA sequence assembly program. Genome research 9, 868–877 (1999). 10508846 10.1101/gr.9.9.868 PMC310812

[b52] Zhang Z. . KaKs_Calculator: calculating Ka and Ks through model selection and model averaging. Genomics, proteomics & bioinformatics 4, 259–263 (2006). 10.1016/S1672-0229(07)60007-2 PMC5054075 17531802

[b53] Szklarczyk D. . The STRING database in 2011: functional interaction networks of proteins, globally integrated and scored. Nucleic Acids Res 39, D561–568, doi: 10.1093/nar/gkq973 (2011). 21045058 PMC3013807

[b54] Kelley L. A., Mezulis S., Yates C. M., Wass M. N. & Sternberg M. J. E. The Phyre2 web portal for protein modeling, prediction and analysis. Nat. Protocols 10, 845–858 (2015). 25950237 10.1038/nprot.2015.053 PMC5298202

[b55] Weirauch M. T. . Determination and inference of eukaryotic transcription factor sequence specificity. Cell 158, 1431–1443 (2014). 25215497 10.1016/j.cell.2014.08.009 PMC4163041

[b56] Van Der Spoel D. . GROMACS: fast, flexible, and free. Journal of computational chemistry 26, 1701–1718 (2005). 16211538 10.1002/jcc.20291

