# Supplementary material for: In Silico identification of SNP diversity in cultivated and wild tomato species: insight from molecular simulations
Source: Sci Rep. 2016 Dec 8;6:38715. doi: 10.1038/srep38715 (PMC5144076; doi:10.1038/srep38715)
Supplement: Supplementary Figures and Tables [file srep38715-s1.pdf]

Supplementary Information

In Silico identification of SNP diversity in cultivated and wild tomato species: insight from molecular simulations

Archana Bhardwaj[a,b], Yogeshwar Vikram Dhar[a,b], Mehar Hasan Asif[b] and Sumit K Bag[a,b,*]

[a]Academy of Scientific and Innovative Research (AcSIR), CSIR-NBRI Campus, Lucknow, India

[b]Council of Scientific and Industrial Research - National Botanical Research Institute (CSIR-NBRI), Rana Pratap Marg, Lucknow-226001, INDIA

The email addresses for all the authors:

Archana Bhardwaj - archana2287@gmail.com

Yogeshwar Vikram Dhar - yogi.bioinfo@gmail.com

Mehar Hasan Asif - mh.asif@nbri.res.in

Sumit K Bag - sumit.bag@nbri.res.in

***Corresponding author:**

Dr. Sumit K Bag, Senior Scientist, CSIR-National Botanical Research Institute, Rana Pratap Marg, Lucknow, INDIA; Ph:+91-522-2297914 **Fax:** +91-522-2205836, 2205839, Email - sumit.bag@nbri.res.in

Running Title - SNPs in wild and cultivated species of tomato

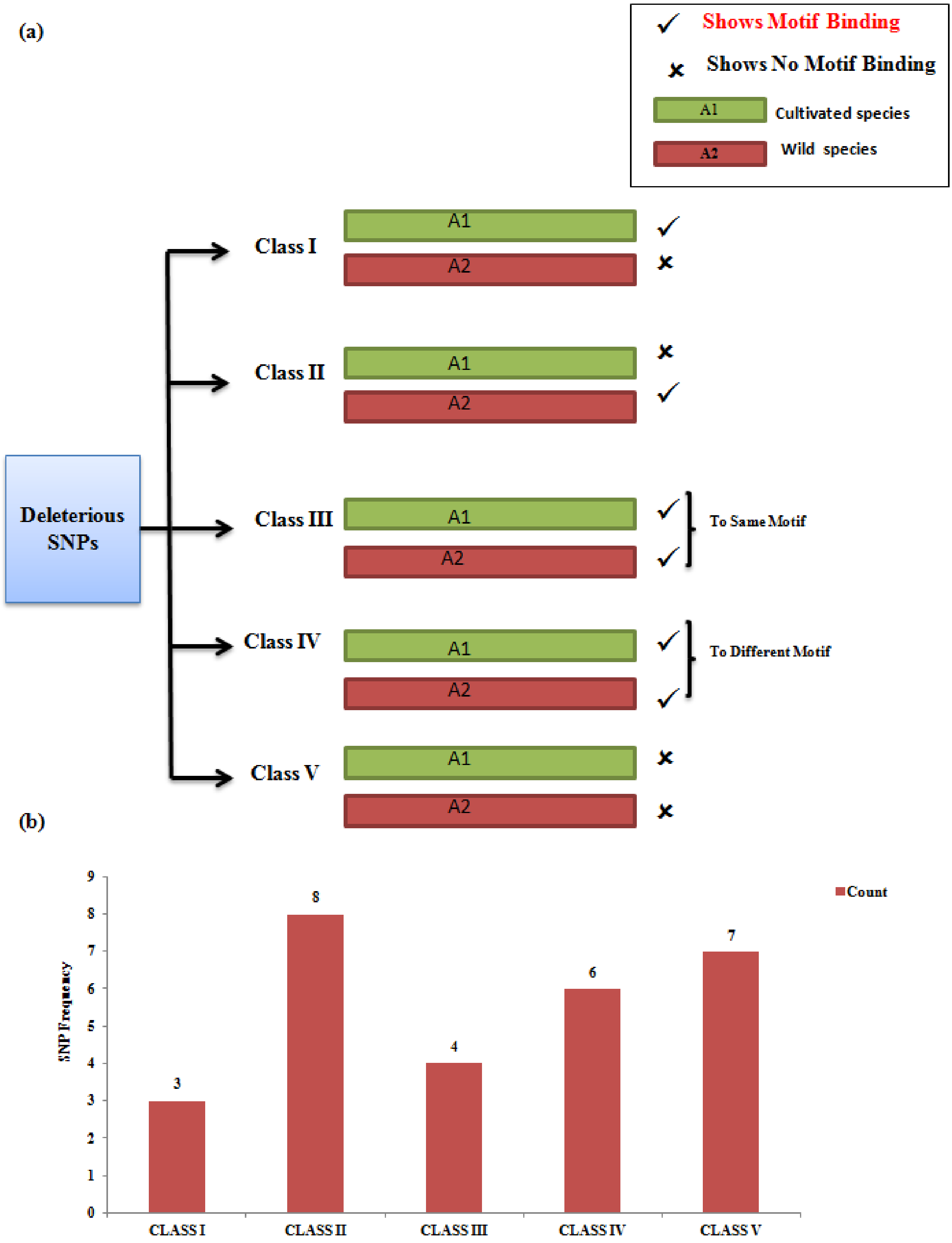

Supplementary Figure S1: ***SNPs distributed in five distinct classes based on the differential motif binding and their SNPs frequency*** (a) Class I consist of depletion in motif

binding due to nucleotide change from cultivated to wild species, class II shows depletion in motif binding from wild to cultivated species, class III represents the binding to same motif (both allele binds to same motif), class IV SNPs consist of change in motif family for both the allele, class V SNPs shows no binding to any motif (b) Frequency of SNPs distributed in five distinct classes.

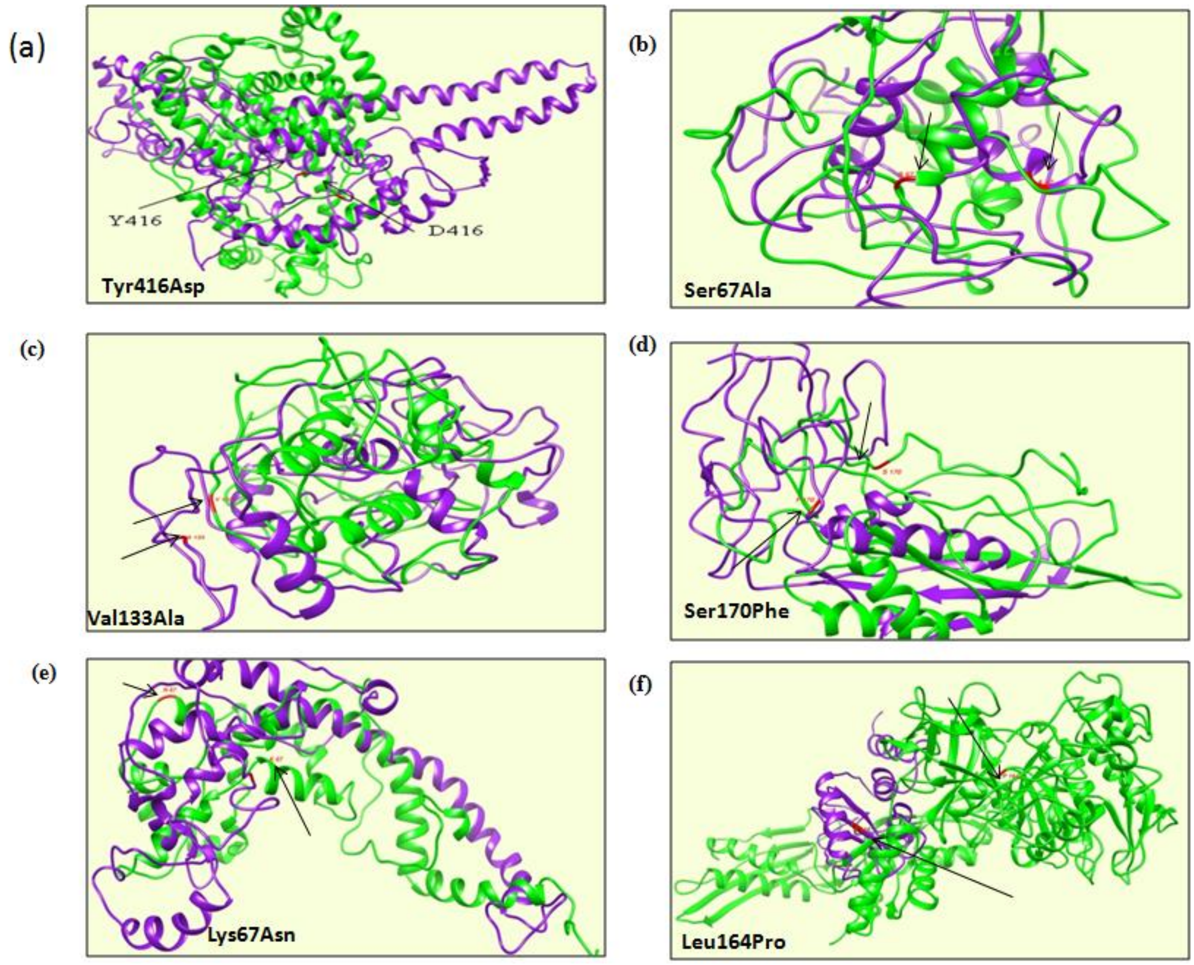

Supplementary Figure S2: ***Structural alignment of multiple Native and associated mutant protein models:*** Superimposed 3D structure of native and mutant protein models of (a) HMG1 (high mobility group 1) gene (b) H1 histone-like gene (c) Histone H1 gene (d) Ribonuclease P gene (e) Reticulon family gene (f) Elongation factor-G gene. Green ribbon represents the native and purple ribbon represents the associated mutant protein models. Arrow indicate the position of SNP or substitution site.

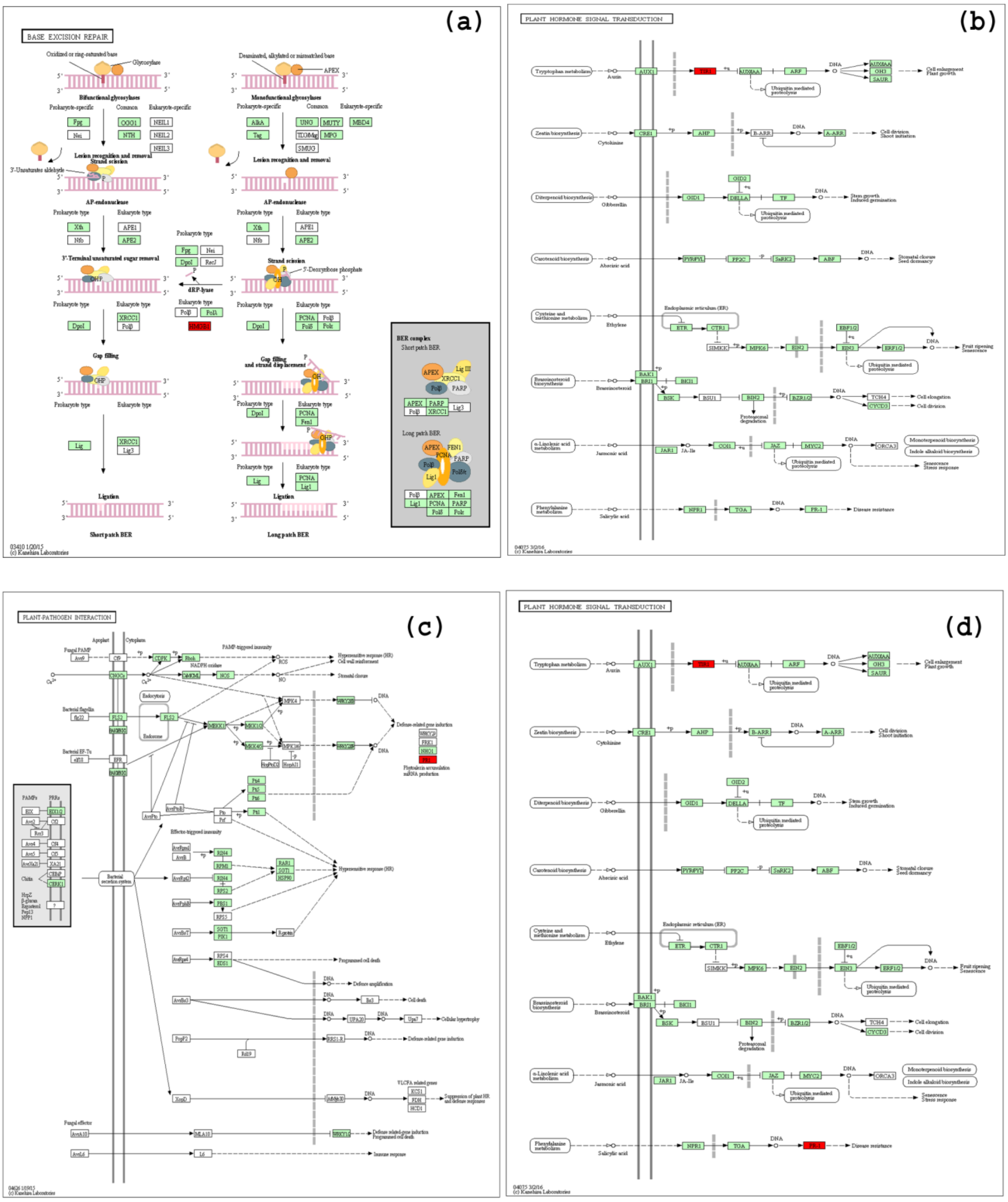

Supplementary Figure S3: ***Involvement of SNPs in multiple KEGG pathways:*** SNPs are involved in (a) HMGI (high mobility group 1) gene of Base exision repair pathway having Asp115Glu substitution (b) T1R1 (pathogen responsive 1) gene of Plant hormone signal having Ser144Cys substitution (c) PR1 (pathogen responsive 1) gene of plant pathogen interaction pathway having Ser145Cys substitution (d) PR-1 (pathogen responsive-1) gene of plant hormone signal transduction pathway having Cys284Arg substitution.

Supplementary Table S1. List of Purified and Diversified Gene groups along with Ka/Ks ratio

Gene	Ka/Ks	Category	Description
Solyc07g064130	0.0268478	Purified	Ubiquitin
Solyc11g066130	0.0308513	Purified	osmotin
Solyc09g010630	0.0317003	Purified	HSC2-like
Solyc11g069090	0.0323036	Purified	ATP-binding cassette protein
Solyc09g092380	0.0327451	Purified	S-adenosyl-l-homocysteine hydrolase
Solyc10g081510	0.0339152	Purified	ethylene-responsive methionine synthase
Solyc02g065090	0.0341827	Purified	Patatin-like protein 3
Solyc12g099970	0.0387128	Purified	SNF1 kinase complex anchoring protein
Solyc03g114340	0.0446837	Purified	Dxr encodes a non-limiting regulatory enzyme
Solyc03g118040	0.0454497	Purified	calnexin
Solyc06g051200	0.0469131	Purified	50S ribosomal protein L3
Solyc08g076220	0.0470112	Purified	Phosphoribulokinase/uridine kinase
Solyc11g069790	0.0474334	Purified	chaperonin
Solyc04g007550	0.0476434	Purified	ATP synthase subunit beta
Solyc07g042250	0.0480481	Purified	chaperonin 21 precursor
Solyc02g082930	0.0486028	Purified	acidic extracellular 27 kD chitinase
Solyc09g011810	0.0488013	Purified	Fructose-1 6-bisphosphatase class 1
Solyc06g035450	0.0494417	Purified	ATP-dependent RNA helicase
Solyc06g050170	0.051884	Purified	Potassium transporter
Solyc02g086820	0.0523366	Purified	chloroplast carbonic anhydrase
Solyc04g080630	0.0523755	Purified	50S ribosomal protein L31
Solyc04g008460	0.0524414	Purified	Ribosomal protein L15
Solyc03g117480	0.0542053	Purified	Pyrophosphate-energized proton pump
Solyc10g077120	0.0553006	Purified	Photosystem II core complex proteins psbY
Solyc10g086580	0.05743	Purified	Ribulose-1 5-bisphosphate carboxylase/oxygenase activase 1
Solyc12g044330	0.057709	Purified	Aquaporin
Solyc05g005710	0.0582398	Purified	spermidine synthase
Solyc01g094950	0.0585515	Purified	Protein BPS1, chloroplastic
Solyc09g082830	0.0587064	Purified	member of the argonaute gene family of tomato
Solyc01g101060	0.0590374	Purified	S-adenosyl-L-methionine synthetase
Solyc09g064500	0.0594228	Purified	Photosystem II reaction center psb28 protein
Solyc04g077510	0.0600072	Purified	Growth regulating factor 1
Solyc09g007560	0.0613897	Purified	50S ribosomal protein L5
Solyc01g112290	0.0619459	Purified	Glutamyl-tRNA synthetase
Solyc01g099900	0.0628734	Purified	Ribosomal protein L18
Solyc09g007250	0.0629964	Purified	60S ribosomal protein L4/L1
Solyc01g103450	0.0634874	Purified	Chaperone DnaK
Solyc07g056540	0.0636372	Purified G	glycolate oxidase X92888

Solyc04g009200	0.0637986	Purified	glutamate 1-semialdehyde 2,1-aminomutase
Solyc07g007600	0.0653518	Purified	vacuolar-type H+-pyrophosphatase
Solyc04g010250	0.0662751	Purified	Lipase-like protein
Solyc03g115200	0.0663311	Purified	Glucan endo-1 3-beta-glucosidase 1
Solyc11g013110	0.067135	Purified	Anthocyanidin synthase
Solyc10g078300	0.0682599	Purified	Single-stranded nucleic acid binding R3H protein
Solyc10g086330	0.069001	Purified	60S ribosomal protein L23a
Solyc05g056350	0.0691355	Purified	Cleft lip and palate associated transmembrane protein-like
Solyc09g006010	0.0692287	Purified	Pathois related protein PR-1
Solyc11g069940	0.0693842	Purified	Glutaredoxin
Solyc07g005510	0.0701973	Purified	Omega-6 fatty acid desaturase
Solyc12g056390	0.0725632	Purified	Thaumatin-like protein
Solyc09g075430	0.0729236	Purified	Ribosomal protein L19
Solyc07g065490	0.0734698	Purified	Dek protein
Solyc01g028860	0.0739126	Purified	YTH domain family 2
Solyc12g095990	0.0746926	Purified	belongs to the helicase gene family
Solyc02g065470	0.0748429	Purified	Pathois-related protein
Solyc08g074620	0.0753707	Purified	Polyphenol oxidase
Solyc11g071620	0.0760739	Purified	member of the aldehyde oxidase gene family known
Solyc11g009080	0.0760866	Purified	DAHP synthase 1 precursor
Solyc11g066390	0.0762184	Purified	chloroplastic Cu,Zn superoxide dismutase
Solyc06g083620	0.0768738	Purified	26S protease regulatory subunit 4
Solyc07g061900	0.0768973	Purified	50S ribosomal protein L4
Solyc02g091880	0.0773238	Purified	Cytochrome c oxidase subunit VIa family protein expressed
Solyc01g105050	0.0780733	Purified	Chlorophyll a-b binding protein, chloroplastic
Solyc02g080540	0.0783086	Purified	ATP synthase gamma chain
Solyc06g054260	0.0784917	Purified	photosystem 1 reaction center protein subunit 2
Solyc12g099440	0.0788108	Purified	Fatty acid oxidation complex subunit alpha
Solyc07g054210	0.0796402	Purified	light dependent NADH:protochlorophyllide oxidoreductase 2
Solyc07g005810	0.0807537	Purified	Eukaryotic translation initiation factor 4
Solyc06g065390	0.0814134	Purified	50S ribosomal protein L21
Solyc05g008600	0.0817077	Purified	ripening regulated protein
Solyc04g054480	0.0822813	Purified	C2 domain-containing protein-like
Solyc01g105410	0.0826583	Purified	Os06g0220000 protein
Solyc07g049370	0.084985	Purified	Glucan endo-1 3-beta-glucosidase A6
Solyc01g091530	0.0857803	Purified	Fasciclin-like arabinogalactan protein 13
Solyc01g009520	0.0858863	Purified	Ribosomal protein
Solyc08g016180	0.0865655	Purified	60S ribosomal protein L18a
Solyc03g121180	0.0871276	Purified	GDSL esterase/lipase At5g22810
Solyc06g035460	0.0891004	Purified	DEAD-box ATP-dependent RNA helicase 7

Solyc11g020960	0.0918754	Purified	Proteinase inhibitor II
Solyc06g073330	0.0919171	Purified	Lysyl-tRNA synthetase
Solyc07g043420	0.0926572	Purified	similar to hyoscyamine 6 beta-hydroxylase
Solyc03g034220	0.0931223	Purified	Tomato RuBP carboxylase small subunit
Solyc01g103540	0.0931528	Purified	YTH domain family 2
Solyc03g096940	0.0933042	Purified	NADH ubiquinone oxidoreductase subunit
Solyc03g113730	0.0943208	Purified	B12D protein
Solyc07g063850	0.0945567	Purified	unknown function
Solyc12g042500	0.0948106	Purified	Gibberellin-regulated family protein
Solyc08g006150	0.0953488	Purified	ChaC cation transport regulator-like 1
Solyc02g081810	0.0955742	Purified	tRNA pseudouridine synthase B
Solyc07g005830	0.0963301	Purified	Translation initiation factor IF-3
Solyc10g086730	0.0966878	Purified	Fructose-1 6-bisphosphatase class 1
Solyc09g018450	0.097221	Purified	Ubiquitin-activating enzyme E1
Solyc08g006530	0.0975232	Purified	CONSTANS-like protein
Solyc09g010840	0.0975404	Purified	member of SlR2R3MYB gene family
Solyc10g006070	0.0977264	Purified	40S ribosomal protein S8
Solyc06g082100	0.0979555	Purified	Related to ATP dependent RNA helicase
Solyc07g043360	0.0983882	Purified	60S ribosomal protein L27
Solyc02g068420	0.0984612	Purified	Glioma tumor suppressor candidate region gene 2
Solyc12g038980	0.0988435	Purified	50S ribosomal protein L7Ae
Solyc10g084350	0.0992038	Purified	60S ribosomal protein L4-B
Solyc06g065680	0.0995089	Purified	SlCycA2.2 is a part of the 52 member cyclin gene family in tomato.
Solyc01g101240	0.0998844	Purified	aspartic protease precursor
Solyc07g044840	0.100847	Purified	2 3-bisphosphoglycerate-independent phosphoglycerate mutase
Solyc09g075830	0.101033	Purified	Time for coffee
Solyc02g070540	0.101601	Purified	Os01g0611000 protein
Solyc07g041310	0.102481	Purified	Ribosomal protein
Solyc11g006780	0.102868	Purified	Vesicle-associated membrane protein 7B
Solyc07g066150	0.103131	Purified	Photosystem I reaction center subunit V
Solyc01g111170	0.103242	Purified	Low sensitivity to the hormone auxin. Stems tend to grow parallel to the soil surface
Solyc02g081160	0.103272	Purified	Diphosphate-fructose-6-phosphate 1-phosphotransferase
Solyc09g075360	0.103468	Purified	endo-1,4-beta-glucanase precursor
Solyc08g078530	0.103486	Purified	Agenet domain containing protein expressed
Solyc07g005100	0.10374	Purified	Chitinase-like protein
Solyc01g098640	0.103851	Purified	UPF0603 protein At1g54780, chloroplastic
Solyc04g074980	0.103882	Purified	Auxin F-box protein 5
Solyc09g092250	0.1039	Purified	SlC3H55 is part of the 90 member Zinc finger transcription factor gene family.
Solyc08g014340	0.104248	Purified	Cysteine synthase

Solyc05g056000	0.104346	Purified	Lipase-like protein
Solyc10g084640	0.104362	Purified	N-acetyltransferase
Solyc02g091840	0.104435	Purified	Receptor-like protein kinase 3
Solyc10g075090	0.1045	Purified	Non-specific lipid-transfer protein
Solyc09g009260	0.105092	Purified	fructose-1,6-bisphosphate aldolase
Solyc02g090030	0.10522	Purified	33 kDa oxygen-evolving protein
Solyc10g006030	0.10592	Purified	30S ribosomal protein S10-like
Solyc01g111450	0.107704	Purified	LEY14339 proteasome, alpha subunit
Solyc12g009250	0.10783	Purified	chaperonin
Solyc01g008960	0.108238	Purified	member of the argonaute gene family of tomato
Solyc04g081200	0.108256	Purified	Regulator of chromosome condensation
Solyc10g008980	0.108611	Purified	triose phosphate translocator
Solyc10g006580	0.10874	Purified	ribosomal protein L2
Solyc06g005710	0.10911	Purified	cDNA clone 002-143-C11 full insert sequence
Solyc08g008230	0.109131	Purified	Arabinogalactan peptide 22
Solyc11g006710	0.109349	Purified	Cationic amino acid transporter
Solyc04g014460	0.110325	Purified	RNA binding protein
Solyc09g076050	0.110573	Purified	FRIGIDA
Solyc04g082010	0.110836	Purified	pre-plastocyanin
Solyc08g081580	0.111964	Purified	Pin2-interacting protein X1
Solyc01g111300	0.114588	Purified	Cold shock protein-1
Solyc11g073250	0.114608	Purified	Histone H2A
Solyc06g053340	0.119365	Purified	ApaG protein
Solyc09g089670	0.120718	Purified	Nicotiana lesion-inducing like
Solyc06g083270	0.122052	Purified	ER lumen retaining receptor family-like protein
Solyc11g040370	0.124645	Purified	Far upstream element-binding protein 3
Solyc10g006290	0.128552	Purified	constitutive plastid-lipid associated protein
Solyc10g080870	0.132112	Purified	Cytochrome P450
Solyc02g077710	0.134046	Purified	E6-2 protein kinase
Solyc06g073180	0.13613	Purified	CONSTANS interacting protein 1
Solyc02g092980	0.136458	Purified	SlCycD3_4/cycd3 is a part of the 52 member cyclin gene family in tomato.
Solyc12g006920	0.138417	Purified	Serine/threonine-protein phosphatase 2A regulatory subunit delta 1 isoform
Solyc02g063150	0.140521	Purified	RuBP carboxylase small subunit
Solyc08g074630	0.140582	Purified	Polyphenol oxidase
Solyc06g060850	0.140877	Purified	Ubiquitin carboxyl-terminal hydrolase isozyme L3
Solyc04g051800	0.140951	Purified	ABC transporter ATP-binding protein
Solyc02g085950	0.142299	Purified	cell wall protein X77373
Solyc11g008270	0.142345	Purified	Genomic DNA chromosome 3 P1 clone MSJ11
Solyc11g005330	0.144145	Purified	actin
Solyc10g055680	0.145328	Purified	Ubiquinone/menaquinone biosynthesis methyltransferase ubiE

Solyc07g047850	0.148695	Purified	unknown function
Solyc08g083110	0.148972	Purified	Cystathionine gamma-lyase
Solyc10g005110	0.149018	Purified	Coproporphyrinogen III oxidase aerobic
Solyc08g061100	0.149754	Purified	Cellulose synthase
Solyc06g073540	0.151729	Purified	member of the argonaute gene family of tomato
Solyc06g053450	0.15228	Purified	SlSUN17 is part of the 34 member SUN-like gene family
Solyc02g092860	0.152428	Purified	Cytochrome P450
Solyc02g067050	0.15272	Purified	Uncharacterized ACR COG1678 family protein
Solyc03g019780	0.153697	Purified	40S ribosomal protein SA
Solyc03g096380	0.155333	Purified	proline transporter
Solyc11g056680	0.155918	Purified	Leucine-rich repeat family protein / protein kinase family protein
Solyc03g096840	0.158457	Purified	Seed specific protein Bn15D1B
Solyc01g067730	0.158534	Purified	Acyl carrier protein
Solyc04g071900	0.158586	Purified	Peroxidase
Solyc08g060810	0.15869	Purified	regulates the ethylene signalling pathway
Solyc02g084550	0.162034	Purified	Chromosome 10 contig 1 DNA sequence
Solyc11g066840	0.163479	Purified	Histone deacetylase-like protein
Solyc08g076670	0.165646	Purified	Transmembrane protein 41B
Solyc03g119130	0.173038	Purified	Histone H1
Solyc02g067530	0.173342	Purified	Prolyl 4-hydroxylase alpha subunit-like protein
Solyc10g078240	0.178324	Purified	Cytochrome P450
Solyc09g072770	0.179197	Purified	mRNA clone RAFL22-93-M12
Solyc03g098280	0.180493	Purified	member of the argonaute gene family
Solyc08g082070	0.180919	Purified	TOX high mobility group box family member 4
Solyc06g036430	0.182006	Purified	Reticulon family protein
Solyc12g011010	0.182694	Purified	Meiosis 5
Solyc12g015880	0.18653	Purified	regulate proper folding of peptides
Solyc11g069150	0.188056	Purified	Proteasome subunit beta type
Solyc06g009400	0.19008	Purified	*piiglb1*
Solyc09g083380	0.199708	Purified	Histone H1
Solyc09g007490	0.20784	Purified	Cell number regulator 8
Solyc07g043390	0.208814	Purified	Cellulose synthase family protein expressed
Solyc10g055630	0.208851	Purified	SlPIP2.9 gene belongs to the 47 member aquaporin gene family.
Solyc01g100570	0.211966	Purified	Nucleolar protein
Solyc03g058920	0.212517	Purified	Porin/voltage-dependent anion-selective channel protein
Solyc02g062340	0.217956	Purified	Fructose-bisphosphate aldolase
Solyc09g010460	0.22151	Purified	Eukaryotic translation initiation factor 3 subunit A
Solyc02g094400	0.221665	Purified	Glycerophosphodiester phosphodiesterase gde1
Solyc04g077490	0.223304	Purified	AP2-like ethylene-responsive transcription factor At1g16060

Solyc09g057650	0.224393	Purified	40S ribosomal protein S8
Solyc04g071800	0.228511	Purified	Cytochrome P450
Solyc11g006250	0.232093	Purified	GDSL-motif lipase/hydrolase family protein 2
Solyc09g090500	0.233883	Purified	Cyclopropane-fatty-acyl-phospholipid synthase
Solyc08g077180	0.235989	Purified	Pyruvate kinase
Solyc09g066100	0.236093	Purified	Histone H1
Solyc03g044200	0.263074	Purified	Alcohol dehydrogenase
Solyc01g081010	0.317437	Purified	Nucleolar GTP-binding protein
Solyc06g059930	0.366587	Purified	β-carophyllene and α-humulene
Solyc06g084050	1.01317	Diversified	Photosystem II reaction center W protein
Solyc02g078040	1.01518	Diversified	Pistil extensin like protein
Solyc11g007070	1.0195	Diversified	Mitochondrial substrate carrier family protein
Solyc05g013010	1.02178	Diversified	Sulfotransferase family protein
Solyc07g038160	1.06027	Diversified	GATA transcription factor 20
Solyc05g025600	1.06363	Diversified	Chloroplast photosystem II subunit X
Solyc07g018070	1.09675	Diversified	Heat shock protein-related
Solyc02g083860	1.11713	Diversified	Flavanone 3-hydroxylase
Solyc11g006230	1.13727	Diversified	Calcium-responsive transactivator
Solyc01g087500	1.13826	Diversified	DNA topoisomerase 2
Solyc01g104470	1.15979	Diversified	mRNA turnover protein 4 homolog
Solyc03g114940	1.16315	Diversified	Cytochrome P450 family protein
Solyc02g085420	1.17649	Diversified	U1 small nuclear ribonucleoprotein
Solyc05g023740	1.1821	Diversified	Arabidopsis mei2-like protein
Solyc10g081440	1.18388	Diversified	NADH cytochrome b5 reductase
Solyc09g084490	1.18988	Diversified	Proteinase inhibitor I
Solyc06g083180	1.20672	Diversified	40S ribosomal protein S8
Solyc11g068430	1.23202	Diversified	Ferredoxin
Solyc12g098890	1.25065	Diversified	50S ribosomal protein L18
Solyc06g083690	1.26661	Diversified	Glutaredoxin
Solyc01g014280	1.30686	Diversified	Unknown Protein
Solyc01g094460	1.37953	Diversified	AT-hook motif nuclear localized protein 1
Solyc08g065420	1.45771	Diversified	bell-like homeodomain protein 4
Solyc06g061140	1.52781	Diversified	Genomic DNA chromosome 5 BAC clone F10E10
Solyc04g005480	1.57615	Diversified	Unknown Protein
Solyc05g008300	1.61739	Diversified	Unknown Protein
Solyc09g082650	1.62171	Diversified	Acireductone dioxygenase
Solyc09g011140	1.79649	Diversified	Tropinone reductase I
Solyc02g078570	1.81697	Diversified	Epoxide hydrolase 3
Solyc07g053280	1.95357	Diversified	Ketol-acid reductoisomerase
Solyc04g077820	2.03914	Diversified	ZCW7 protein
Solyc03g114740	2.09512	Diversified	BSD domain containing protein
Solyc12g009650	2.19581	Diversified	Sl proline-rich protein

Solyc05g054120	4.16233	Diversified	Cysteine proteinase inhibitor

Supplementary Table S2: Deleterious SNPs found as active site

Gene ID	AA change	Active site Prediction	Description
Solyc02g084240	Ser67Ala	YES	H1 histone-like protein involved in DNA binding
Solyc04g081880	Ser170Phe	YES	Ribonuclease P protein subunit p25
Solyc05g006730	Asp105Asn	YES	Glutathione S-transferase 3
Solyc06g036430	Lys67Asn	YES	Reticulon family protein
Solyc01g090690	Leu164Pro	YES	Elongation factor G
Solyc09g066100	Val133Ala	YES	Histone H1
Solyc02g082700	Asp115Glu	YES	High mobility group protein
Solyc08g082070	Tyr416Asp	YES	TOX high mobility group box family member 4
Solyc09g011140	Asn150Tyr	NO	Tropinone reductase I
Solyc01g111450	Glu91Gly	NO	LEY14339 proteasome, alpha subunit
Solyc02g080810	Leu250Phe	NO	Aminomethyltransferase
Solyc04g011400	Gly315Arg	NO	UDP-glucose 4-epimerase
Solyc04g074980	Ser270Cys	NO	Auxin F-box protein 5
Solyc04g081850	Gly155Ala	NO	UPF0436 protein C9orf6 homolog
Solyc05g018810	Lys71Asn	NO	Nucleosome assembly protein 1-like protein 2
Solyc05g056000	Leu302Pro	NO	Lipase-like protein
Solyc05g056000	Lys289Arg	NO	Lipase-like protein
Solyc05g056390	Asn32Lys	NO	chaperonin
Solyc06g036050	Leu42Pro	NO	60S ribosomal protein L36
Solyc07g014700	Leu588Pro	NO	Dolichyl-diphosphooligosaccharide--protein glycosyltransferase subunit 2
Solyc08g005680	Glu140Gly	NO	zFPS encodes a protein, with homology to Z-isoprenyl pyrophosphate synthase, that catalyzes the synthesis of Z,Z-FPP from IPP and DMAPP. zFPS colocalizes with SBS at the Sst2 locus. Co-expression of both genes results in the synthesis of
Solyc08g006330	Ile343Met	NO	UDP-xylose phenolic glycosyltransferase
Solyc01g099410	Leu103Met	NO	Histone H2A
Solyc06g083270	Cys254Arg	NO	ER lumen retaining receptor family-like protein
Solyc09g007020	Ser145Cys	NO	Pathogenesis-related protein
Solyc09g074520	Cys284Arg	NO	Auxin F-box protein 5
Solyc00g174340	Ser144Cys	NO	Pathogenesis-related protein 1b
Solyc02g091280	Ile120Phe	NO	ATP-dependent Clp protease proteolytic subunit

Supplementary Table S3: Differential binding of fruit ripening, fruit texture and cold tolerant genes

Gene ID	Position	Allele 1	Allele 2	Allele	Name	MotifID	Family	From	To	Regulatory Process
Solyc01g087210	3276/4052	T	A	A2 only	ZAT7(T037032_1.02)	M0373_1.02	C2H2 ZF	3	11	texture
Solyc01g087210	2437/4052	C	T	A2 only	ATHB7(T090224_1.02)	M1108_1.02	Homeodomain	5	13	texture
	2437/4052	C	T	A2 only	GATA11(T084285_1.02)	M0759_1.02	GATA	9	18	texture
	2437/4052	C	T	A2 only	AT4G29000(T073820_1.02)	M0591_1.02	CxC	3	11	texture
	2437/4052	C	T	A2 only	GATA10(T084284_1.02)	M0758_1.02	GATA	10	18	texture
	2437/4052	C	T	A2 only	ATWOX13(T090250_1.02)	M0853_1.02	Homeodomain	5	13	texture
	2437/4052	C	T	A2 only	ATML1(T090244_1.02)	M0852_1.02	Homeodomain	10	18	texture
	2437/4052	C	T	A1 only	AT2G21230(T023835_1.02)	M0260_1.02	bZIP	8	16	texture
Solyc01g091530	1253/1634	A	G	A1 only	MYB24(T116988_1.02)	M1343_1.02	Myb/SANT	5	13	texture
	1253/1634	A	G	A2 only	ASIL1(T148732_1.02)	M1618_1.02	Storekeeper	6	14	texture
Solyc02g065380	652/1130	A	C	A2 only	E2F1(T076547_1.02)	M0677_1.02	E2F	10	18	cold
	652/1130	A	C	A2 only	ATMYB4(T116944_1.02)	M1335_1.02	Myb/SANT	3	11	cold
Solyc06g007130	355/1334	A	G	A1 only	ATMYB4(T116944_1.02)	M1335_1.02	Myb/SANT	8	16	cold
	355/1334	A	G	A1 only	AZF1(T037063_1.02)	M0378_1.02	C2H2 ZF	11	19	cold
	355/1334	A	G	A1 only	GT-1(T109447_1.02)	M1291_1.02	MADF	10	18	cold
	355/1334	A	G	A1 only	RHL41(T037058_1.02)	M0377_1.02	C2H2 ZF	11	19	cold
	355/1334	A	G	A1 only	AT3G53600(T037036_1.02)	M0374_1.02	C2H2 ZF	11	19	cold
Solyc07g043390	1360/1922	G	C	A2 only	AT5G05090(T116953_1.02), AT3G10760(T116863_1.02)	M1337_1.02	Myb/SANT	5	18	texture
	1360/1922	G	C	A2 only	GATA11(T084285_1.02)	M0759_1.02	GATA	3	18	texture
	1360/1922	G	C	A2 only	ARR14(T116801_1.02)	M1324_1.02	Myb/SANT	5	18	texture
	1360/1922	G	C	A2 only	ARR1(T116876_1.02)	M1327_1.02	Myb/SANT	5	18	texture
	1360/1922	G	C	A2 only	ARR2(T116923_1.02)	M1384_1.02	Myb/SANT	5	18	texture
	1360/1922	G	C	A2 only	GATA8(T084298_1.02)	M0765_1.02	GATA	3	18	texture

	1360/1922	G	C	A2 only	GATA4(T084299_1.02)	M0766_1.02	GATA	5	18	texture
	1360/1922	G	C	A2 only	GATA10(T084284_1.02)	M0758_1.02	GATA	3	18	texture
	1360/1922	G	C	A2 only	TIFY2B(T084293_1.02)	M0762_1.02	GATA	3	18	texture
	1360/1922	G	C	A2 only	GATA7(T084306_1.02)	M0770_1.02	GATA	5	18	texture
	1360/1922	G	C	A2 only	ARR18(T117010_1.02)	M1345_1.02	Myb/SANT	5	17	texture
	1360/1922	G	C	A2 only	GATA6(T084297_1.02)	M0764_1.02	GATA	5	18	texture
	1360/1922	G	C	A2 only	TIFY2A(T084286_1.02)	M0760_1.02	GATA	5	18	texture
	1360/1922	G	C	A2 only	GATA3(T084305_1.02)	M0769_1.02	GATA	3	18	texture
	1360/1922	G	C	A2 only	GATA1(T084294_1.02)	M0763_1.02	GATA	6	18	texture
	1360/1922	G	C	A2 only	AT3G10113(T116858_1.02), EPR1(T116752_1.02)	M1317_1.02	Myb/SANT	5	18	texture
	1360/1922	G	C	A2 only	LHY(T116733_1.02)	M1365_1.02	Myb/SANT	5	17	texture
	1360/1922	G	C	A2 only	AT4G01280(T116914_1.02), AT3G09600(T116857_1.02)	M1351_1.02	Myb/SANT	5	17	texture
	1360/1922	G	C	A2 only	GATA26(T084301_1.02)	M0767_1.02	GATA	6	18	texture
	1360/1922	G	C	A2 only	GATA27(T084310_1.02)	M0771_1.02	GATA	6	18	texture
Solyc07g043390	718/1922	G	C	A2 only	bZIP68(T023815_1.02)	M0255_1.02	bZIP	9	18	texture
	718/1922	G	C	A2 only	GBF3(T023845_1.02)	M0264_1.02	bZIP	9	17	texture
	718/1922	G	C	A2 only	LRL1(T012398_1.02)	M0250_1.02	bHLH	6	15	texture
	718/1922	G	C	A2 only	GBF2(T023860_1.02)	M0266_1.02	bZIP	9	17	texture
	718/1922	G	C	A2 only	GBF1(T023868_1.02)	M0268_1.02	bZIP	9	17	texture
	718/1922	G	C	A2 only	ZCW32(T012378_1.02)	M0153_1.02	bHLH	6	15	texture
	718/1922	G	C	A2 only	ILR3(T012489_1.02)	M0167_1.02	bHLH	6	15	texture
	718/1922	G	C	A2 only	MYC2(T012373_1.02)	M0152_1.02	bHLH	6	15	texture
	718/1922	G	C	A2 only	AtbZIP63(T023881_1.02)	M0359_1.02	bZIP	9	17	texture
	718/1922	G	C	A2 only	BZIP17(T023842_1.02)	M0263_1.02	bZIP	9	17	texture
	718/1922	G	C	A2 only	AT1G01260(T012351_1.02)	M0150_1.02	bHLH	6	14	texture
	718/1922	G	C	A2 only	MYC3(T012482_1.02)	M0165_1.02	bHLH	6	14	texture
	718/1922	G	C	A2 only	MYC4(T012450_1.02)	M0160_1.02	bHLH	6	14	texture
	718/1922	G	C	A2 only	BEE2(T012465_1.02)	M0161_1.02	bHLH	6	14	texture
Solyc08g061100.2	2201/3759	C	T	A2 only	ATHB7(T090224_1.02)	M1108_1.02	Homeodomain	8	16	texture

Solyc10g045240	671/1539	A	T	A1 only	AT3G10113(T116858_1.02), EPR1(T116752_1.02)	M1317_1.02	Myb/SANT	5	13	fruit ripening
	671/1539	A	T	A1 only	TBP2(T150468_1.02)	M1641_1.02	TBP	4	12	fruit ripening
	671/1539	A	T	A2 only	HB-1(T090196_1.02)	M0841_1.02	Homeodomain	4	18	fruit ripening
	671/1539	A	T	A2 only	AT4G21895(T008609_1.02)	M0126_1.02	AT hook	5	13	fruit ripening
	671/1539	A	T	A2 only	ATML1(T090244_1.02)	M0852_1.02	Homeodomain	4	12	fruit ripening
	671/1539	A	T	A2 only	AT4G17950(T008608_1.02)	M0137_1.02	AT hook	5	13	fruit ripening
Solyc04g011400	1100/1371	C	T	A2 only	GATA11(T084285_1.02)	M0759_1.02	GATA	6	14	fruit ripening
Solyc08g060810	1153/2674	C	A	A1 only	ATTRB2(T117025_1.02)	M1346_1.02	Myb/SANT	4	17	fruit ripening
	1153/2674	C	A	A1 only	TRFL5(T116747_1.02)	M1316_1.02	Myb/SANT	4	17	fruit ripening
Solyc09g074520	1022/2150	T	C	A1 only	AZF1(T037063_1.02)	M0378_1.02	C2H2 ZF	8	19	cold
	1022/2150	T	C	A2 only	E2F1(T076547_1.02)	M0677_1.02	E2F	9	17	cold
	1022/2150	T	C	A1 only	AZF2(T037027_1.02)	M0372_1.02	C2H2 ZF	8	16	cold
	1022/2150	T	C	A1 only	RHL41(T037058_1.02)	M0377_1.02	C2H2 ZF	8	18	cold
	1022/2150	T	C	A1 only	AT3G53600(T037036_1.02)	M0374_1.02	C2H2 ZF	10	18	cold
	1022/2150	T	C	A1 only	STZ(T037008_1.02)	M0370_1.02	C2H2 ZF	8	16	cold
